# Prion infection modulates hematopoietic stem/progenitor cell fate through cell-autonomous and non-autonomous mechanisms

**DOI:** 10.1038/s41375-023-01828-w

**Published:** 2023-01-27

**Authors:** Hyun-Jaung Sim, Yong-Chan Kim, Govinda Bhattarai, Sae-Young Won, Jeong-Chae Lee, Byung-Hoon Jeong, Sung-Ho Kook

**Affiliations:** 1 Department of Bioactive Material Sciences, Research Center of Bioactive Materials, Jeonbuk National University, Jeonju, 54896 Republic of Korea; 2 Cluster for Craniofacial Development and Regeneration Research, Institute of Oral Biosciences and School of Dentistry, Jeonbuk National University, Jeonju, 54896 Republic of Korea; 3 Korea Zoonosis Research Institute, Jeonbuk National University, Iksan, 54531 Republic of Korea

**Keywords:** Haematopoietic stem cells, Infectious diseases

## Abstract

Studies of PrP^C^-derived prion disease generally focus on neurodegeneration. However, little is known regarding the modulation of hematopoietic stem progenitor cells (HSPCs) that express PrP^C^ in prion infection. Among bone marrow (BM) hematopoietic cells, hematopoietic stem cells (HSCs) strongly express PrP^C^. A bioassay revealed the presence of misfolded prion protein (PrP^Sc^) in BM cells derived from prion-infected mice; these BM cells demonstrated reproducible prion infectivity. At 5 months after infection with ME7, mice exhibited a significant decrease in the number of HSPCs. This decrease was mainly driven by increased apoptotic cell death, rather than cell cycle progression and senescence, in PrP^C^-positive but not PrP^C^-negative HSPC populations through a cell-autonomous mechanism. Notably, both PrP^C^-positive and PrP^C^-negative HSCs underwent cellular senescence, as indicated by high levels of senescence-associated factors and deficits in repopulation and self-renewal capacities at 7 months after infection. Senescence of HSCs occurred in the ME7-impaired BM microenvironment with aging phenotypes through non-cell autonomous mechanisms. These data provide novel evidence that prion infection differentially modulates HSC fate through both cell-autonomous and non-autonomous mechanisms.

## Introduction

Prion diseases are a group of fatal neurodegenerative diseases. The major histopathological feature of prion disease is the accumulation of misfolded prion protein (termed PrP^Sc^), which is derived from the structural and conformational alteration of endogenous normal prion protein (termed PrP^C^) [1–3]. Prion diseases have a broad host range, including Creutzfeldt–Jakob disease (CJD) in humans, scrapie in sheep and goats, and bovine spongiform encephalopathy in cattle [4–6]. Under normal physiological conditions, PrP^C^ is predominantly expressed in the brain and plays roles in anti-stress pathways, cellular differentiation, myelin maintenance, and metal ion homeostasis [7]. However, for prion diseases, PrP^C^ is a prerequisite for the impacts of PrP^Sc^. In the absence of PrP^C^, PrP^C^ knockout animals cannot propagate PrP^Sc^ [8, 9].

Although PrP^C^ is strongly expressed in the tissues of the central nervous system, detectable levels of variant CJD prions have recently been observed in the bone marrow (BM), which is an important site that regulates the hematopoietic system through the maintenance of HSPCs [10, 11]. In addition, BM collected from sporadic CJD patients has demonstrated infectivity in human PrP transgenic mice, while BM stromal cells can be infected with a mouse-adapted bovine spongiform encephalopathy strain and demonstrate sporadic CJD [12, 13]. Various hematopoietic cells in the BM and lymph organs express PrP^C^, which is associated with the differentiation of those cells [14]. Moreover, PrP^C^ expression is observed in CD34^+^ stem/progenitor cell populations in human BM; it is also observed in murine CD43^+^B220^-^IL-7R^-^ cell-enriched immature progenitors [15, 16]. Notably, PrP^C^-expressing cells are more abundant among immature long-term repopulating HSC (Lin^-^Sca-1^+^Endoglin^+^ cells) than among other cell types in mouse BM [17]. The role of PrP^C^ in regulating the self-renewal capacity of Lin^-^Sca-1^+^Endoglin^+^ cells has been demonstrated using *Prnp* (prion protein) knockout mice. These findings suggest that the BM can be contaminated with PrP^Sc^ in several prion diseases; furthermore, PrP^C^-expressing mature and immature hematopoietic cells are susceptible to such contamination, with detrimental effects on hematopoietic homeostasis [17]. Although the expression and roles of PrP^C^ have been revealed in various hematopoietic cells, little is currently known regarding the PrP^Sc^-mediated modulation of HSPC fate in prion-infected mice.

Here, we investigated the role of PrP^C^ in HSPC fate, using an animal model of prion disease based on intraperitoneal (i.p.) injection of scrapie strain ME7 into C57BL/6 mice and *Prnp* knockout mice. We provide novel evidence that prion infection differentially modulates HSPC fate through both autonomous and non-autonomous mechanisms that depend on the time since infection.

## Materials and methods

### Animals

Mice were housed (*n* = 5 per cage) under controlled temperature (18–22 °C) and humidity (40–60%) with a 12-h light/dark cycle; food and water were provided ad libitum. C57BL/6J mice (6 weeks old) were purchased from Nara Biotech (Pyeongtaek, Gyeonggi, Korea). *Prnp* (encoding PrP^C^) knockout mice on the FVB background were purchased from The Jackson Laboratory (Bar Harbor, ME, USA). Detailed information regarding the mice was previously published [3]. Equal numbers of male and female mice were included in each group.

### Genotyping of *Prnp* knockout mice

Genomic DNA was extracted from the tail of each mouse using the QIAamp DNA Blood and Tissue Kit (Qiagen, Hilden, Germany), in accordance with the manufacturer’s protocol. The wild-type and mutant alleles of the *Prnp* gene were amplified from genomic DNA using the specific primer sets listed in Supplementary Table 1.

### Statistical analyses

All data are expressed as means ± standard deviations (SDs). Student’s *t* test was used to determine significant differences between two sets of data; one-way analysis of variance was used for multiple comparisons. All analyses were performed in SPSS software, version 16.0 (Chicago, IL, USA). *P* < 0.05 was considered indicative of statistical significance.

## Results

### PrP^C^ expression in BM-conserved hematopoietic cells and prion infectivity in BM cells

**Fig. 1:**
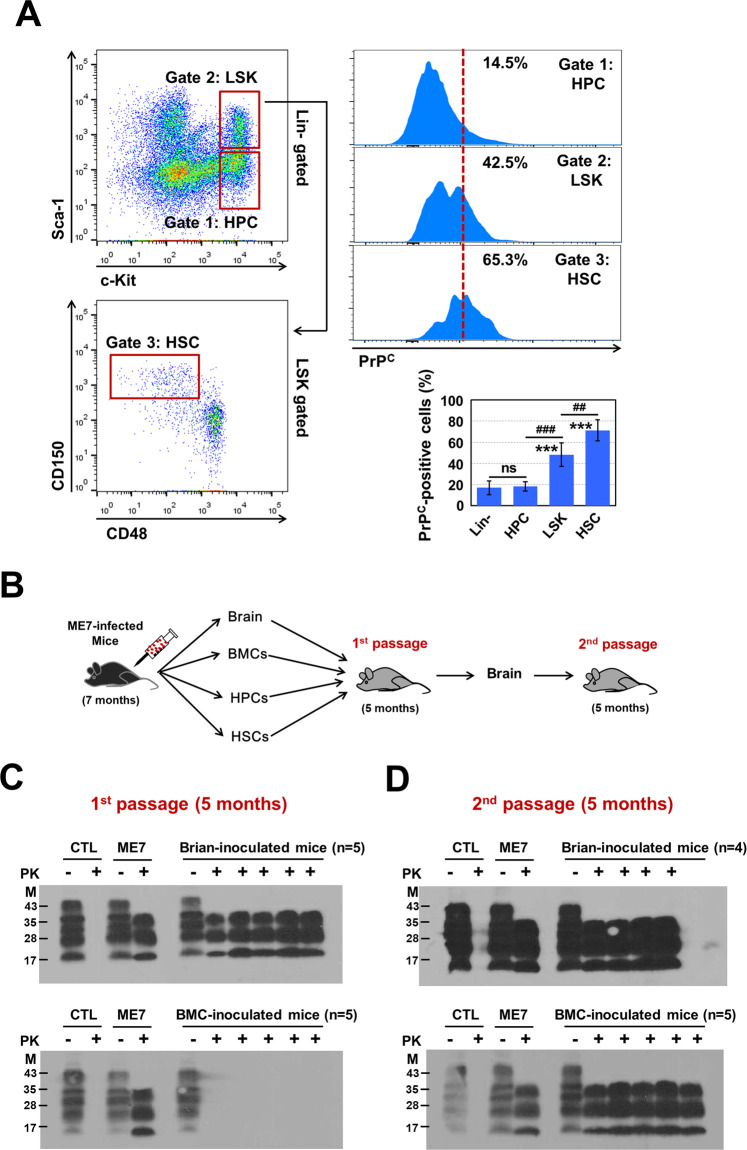
PrP^C^ expression in BM-conserved hematopoietic cells and prion infectivity in BM cells. **A** LSK cells comprise HSCs and a heterogeneous mix of multipotent committed progenitors. LSK cells can be further subdivided into primitive stem cell populations based on the expression patterns of CD150 and CD48 (hereafter referred to as HSCs). HPCs exhibit the following expression pattern: Lin^-^Sca-1^-^c-Kit^+^. PrP^C^ expression in phenotypically defined hematopoietic cells in the BM was examined using a biotinylated PrP^C^ antibody (*n* = 5). **B** Experimental design for the bioassay of prion infectivity using brain tissue and hematopoiesis-related cells such as BMCs, HPCs and HSCs derived from ME7-infected mice. **C** Bioassay results from first-passage mice at 5 months post-injection. The first panel shows western blot bands of PrP^Sc^ in homogenates of brain tissue from mice that had been inoculated with homogenates of brain tissue from prion-infected mice (*n* = 5). The second panel shows western blot bands of PrP^Sc^ in homogenates of brain tissue from mice that had been inoculated with BM cells from prion-infected mice (*n* = 5). The third panel shows western blot bands of PrP^Sc^ in homogenates of brain tissue from mice that had been inoculated with HPCs from prion-infected mice (*n* = 5). The fourth panel shows western blot bands of PrP^Sc^ in homogenates of brain tissue from mice that had been inoculated with HSCs from prion-infected mice (*n* = 4). **D** Bioassay results of second-passage mice at 5 months post-injection. The first panel shows western blot bands of PrP^Sc^ in homogenates of brain tissue from second-passage mice that had been inoculated with homogenates of brain tissue from first-passage mice, which had been inoculated with homogenates of brain tissue from prion-infected mice (*n* = 4). The second panel shows western blot bands of PrP^Sc^ in homogenates of brain tissue from second-passage mice that had been inoculated with homogenates of brain tissue from first-passage mice, which had been inoculated with BM cells from prion-infected mice (*n* = 5). The third panel shows western blot bands of PrP^Sc^ in homogenates of brain tissue from second-passage mice that had been inoculated with homogenates of brain tissue from first-passage mice, which had been inoculated with HPCs from prion-infected mice (*n* = 5). The fourth panel shows western blot bands of PrP^Sc^ in homogenates of brain tissue from second-passage mice that had been inoculated with homogenates of brain tissue from first-passage mice, which had been inoculated with HSCs from prion-infected mice (*n* = 5). All data are presented as means ± SDs. **p < 0.01, and ***p < 0.001 vs. control, as determined by Student’s *t* test and one-way analysis of variance for multiple comparisons using SPSS software (ver.12.0).

To determine how prion infection modulates HSPCs in mice, we assessed the expression level of PrP^C^ protein—the structure of which undergoes conformational alteration to PrP^Sc^ during infection—in hematopoietic cells from the BM. The gating strategy for phenotypic definitions of various hematopoietic stem and progenitor populations is shown in Fig. 1A [18, 19]. Approximately 15% of total BM cells expressed PrP^C^ protein (Supplementary Fig. S1A). The expression level of PrP^C^ gradually increased depending on more immature hematopoietic cells. HSCs exhibited greater expression of PrP^C^ than did LSK cells, which exhibited greater expression than did HPCs or lineage-negative cells (Fig. 1A). These findings suggest that hematopoietic cells in the BM express PrP^C^ protein at various levels; additionally, PrP^C^-positive cells are more enriched among primitive HSCs than among other hematopoietic cell types.

To investigate hematopoietic cell-related properties in prion diseases, we constructed a mouse model (6 weeks of age) of prion disease through i.p. injection of the ME7 scrapie strain; we then measured the body weight of ME7-infected mice at three time points. A significant reduction in the body weight of infected mice was observed at 7 months post-injection, after which the infected mice exhibited abnormal behaviors such as physical inactivity, shivering, and loss of balance, which differed from mice at 5 months post-infection and corresponding control mice (Supplementary Fig. S1B and Supplementary Video S1). All infected mice died before 8 months post-infection (data not shown).

We next investigated the ME7 infection-associated conformational alteration of PrP^C^ to PrP^Sc^. Homogenates of brain tissue from control mice did not exhibit PrP^Sc^ bands, whereas homogenates of brain tissue from ME7-infected mice showed bands at 5 and 7 months, but not at 3 months, post-injection (Supplementary Fig. S1C, upper gel). Although western blotting did not reveal PrP^Sc^ bands in the BM cells from ME7-infected mice (Supplementary Fig. S1C, lower gel), the expression of PrP^Sc^ was increased in BM cells from infected mice at 5 and 7 months post-injection (Supplementary Fig. S1D).

To evaluate prion infectivity in hematopoiesis-related cells, we conducted a bioassay (Fig. 1B). In the first passage, PrP^Sc^ bands were detected in homogenates of brain tissue from mice that had been inoculated with homogenates of brain tissue from infected mice. However, no PrP^Sc^ bands were detected in homogenates of brain tissue from mice that had been inoculated with homogenates of BM cells, HPCs, or HSCs (Fig. 1C and Supplementary Fig. S2A). Notably, PrP^Sc^ bands were detected in homogenates of brain tissue from second-passage mice that had been inoculated with homogenates of brain tissue from first-passage mice, which had been inoculated with BM cells from ME7-infected mice (Fig. 1D, second panel). However, no PrP^Sc^ bands were detected in homogenates of brain tissue from second-passage mice that had been inoculated with homogenates of brain tissue from first-passage mice, which had been inoculated with HPCs or HSCs from ME7-infected mice (Supplementary Fig. S2). This finding may be attributed to the presence of trace amounts of transformed PrP^Sc^ in the HPCs and HSCs from ME7-infected mice. Our results demonstrate that the ME7 scrapie strain used in the present study is appropriate for generating a mouse model of prion disease via structural changes from PrP^C^ to PrP^Sc^, as indicated in the brain and BM; moreover, ME7-infected mice beyond 5 months post-injection provide a reasonable mouse model for investigating how ME7 infection directly affects PrP^C^-expressing HSPCs.

### ME7 infection causes a deficit in BM HSPC function

Mice infected for 5 months with ME7 (hereafter referred to as ME7-infected middle-aged mice) exhibited normal PB counts (Supplementary Fig. S3A and B). However, BM cellularity was significantly reduced in infected middle-aged mice, compared to control mice (Supplementary Fig. S3C). These results suggest that ME7 infection affects the BM, but not hematopoiesis, at 5 months post-infection.

**Fig. 2:**
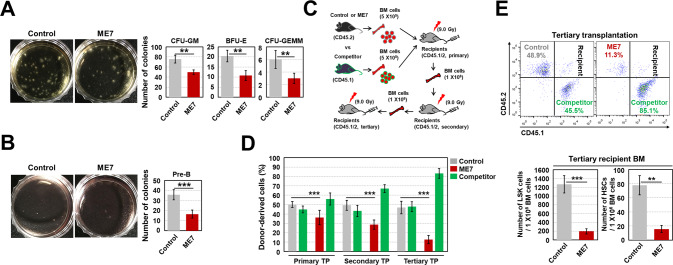
ME7 infection causes functional attrition of BM HSPCs. **A** For colony assays such as colony forming unit-granulocyte-macrophages (CFU-GM), burst -forming nunit-erythroid cells (BFU-E), and CFU-granulocytes, erythroid cells, macrophages, and megakaryocytes (GEMM), BM cells (2 × 10^4^ per dish) from ME7-infected middle-aged mice were incubated in MethoCult GF M3434 medium for 12 days, then subjected to colony counting. Representative data from three independent experiments are shown. **B** For the pre-B colony-forming assay, BM cells (2 × 10^5^ per dish) from ME7-infected mice were incubated in MethoCult M3630 medium for 7 days, then subjected to colony counting. Representative data from three independent experiments are shown. **C**, **D** For analyses of long-term competitive reconstitution capacity and self-renewal capacity, BM cells from control or ME7-infected middle-aged mice (CD45.2) were co-transplanted with equal numbers (5 × 10^5^) of BM cells from competitor mice (CD45.1) into lethally irradiated recipient mice (CD45.1/2, 900 rads, *n* = 7); serial transplantation of BM cells (1 × 10^6^) from CD45.1/2 mice was performed after primary transplantation. The CD45.1/CD45.2 ratio in PB collected from recipient mice at 4 months post-transplantation was evaluated by flow cytometry. **E** Donor-derived repopulation capacity and HSPC engraftment in the PB and BM, respectively, of tertiary recipient mice transplanted with BM cells from secondary recipient mice were assessed via flow cytometry (*n* = 6). All data are presented as means ± SDs. **p < 0.01, and ***p < 0.001 vs. control, as determined by Student’s *t* test.

Based on the above results, we next evaluated HSPC function using a colony-forming assay. BM cells from ME7-infected mice had significantly reduced capacity to establish CFU-GM, CFU-GEMM, BFU-E, and pre-B cells (Fig. 2A, B).

To further assess HSPC function, we conducted competitive repopulation (Fig. 2C). The results showed that similar repopulation capacities of control BM cells and competitor BM cells were observed over a period of serial transplantation (Fig. 2D). In contrast, BM cells derived from ME7-infected mice were outcompeted by BM cells derived from competitor mice after primary transplantation. The poor repopulation performance of infected BM cells in recipient mice became progressively more pronounced with serial transplantation (Fig. 2D). ME7-infected BM cells exhibited defective reconstitution capacity, with a noticeable reduction in the number of ME7-infected mouse-derived HSPCs engrafted in tertiary recipient mice compared to the control mouse-derived counterparts (Fig. 2E). Taken together, these results suggest that ME7 infection leads to loss of functional HSPCs.

### ME7 infection leads to cell-autonomous apoptotic cell death in PrP^C^-positive HSPCs

To investigate the loss of functional HSPCs in ME7-infected middle-aged mice, we measured the frequencies of BM HSPCs. BM cells from infected mice contained significantly smaller numbers of LSK cells and HSCs than did BM cells from control mice (Supplementary Fig. S4A). The numbers of GMP, CMP, and MEP cells did not differ between ME7-infected mice and control mice (Supplementary Fig. S4B) [20]. However, a significantly lower number of CLP cells was observed in ME7-infected mice (Supplementary Fig. S4C) [21]. These results suggest that ME7 infection reduces HSPC abundance.

**Fig. 3:**
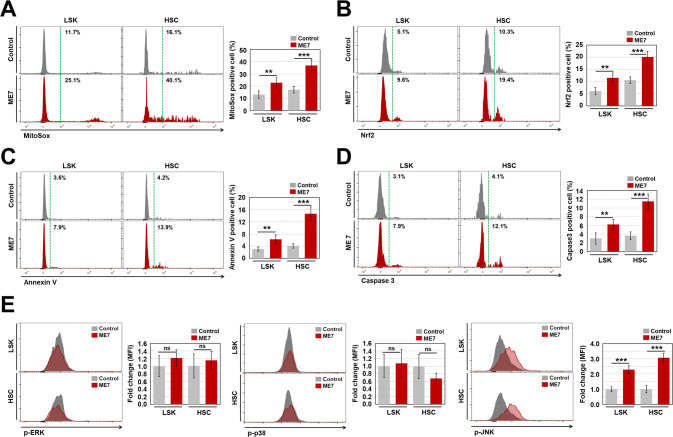
ME7 infection causes apoptotic cell death in PrP^C^-positive HSPCs. **A** Levels of mitochondrial superoxide anions in BM LSK cells and HSCs from middle-aged control and ME7-infected mice were assessed via flow cytometry with MitoSox Red (*n* = 7). **B** Levels of Nrf2 protein were measured in BM LSK cells and HSCs from both mouse groups after cells had been fixed and permeabilized (*n* = 7). Levels of apoptotic cell death in BM LSK cells and HSCs from both mouse groups were analyzed by determination of annexin V (**C**, *n* = 7) and caspase 3 (**D**, *n* = 7). **E** Phosphorylation levels of MAPK proteins (e.g., extracellular signal-regulated kinase [ERK], p38, and JNK) were measured in BM LSK cells and HSCs from both mouse groups after cells had been fixed and permeabilized (*n* = 7). All data are presented as means ± SDs. **p < 0.01, and ***p < 0.001 vs. control, as determined by Student’s *t* test.

We next investigated the causes of the observed decreases in LSK cells and HSCs in ME7-infected middle-aged mice. Oxidative stress functions as a signal trigger that modulates cell proliferation, senescence, and death; [22] therefore, we examined mitochondrial ROS levels in LSK cells and HSCs. The results showed that mitochondrial ROS levels in LSK cells and HSCs were significantly greater in ME7-infected mice than in control mice (Fig. 3A). The levels of nuclear factor erythroid 2-related factor 2 (Nrf2) [23], an emerging regulator of cellular resistance to oxidative stress, in LSK cells and HSCs were significantly greater in ME7-infected mice than in control mice (Fig. 3B).

HSPCs, which exist in a noncycling quiescent state, undergo premature exhaustion and senescence when an excessive proportion of HSPCs enter active cell cycling because of prolonged repopulation demands under stressful conditions [24]. Therefore, we measured the cell cycle profile of HSPCs using an antibody specific for Ki-67, which is a marker expressed in proliferative cells. The results showed no alteration in the percentages of Ki-67-positive LSK cells and HSCs between control and ME7-infected middle-aged mice (Supplementary Fig. S5A).

Because increased levels of ROS are also associated with the induction of aging in stem cells, we next examined the extent of senescence in LSK cells and HSCs from ME7-infected middle-aged mice by measuring senescence-related factors. SA-β-gal activities and mRNA levels of cyclin-dependent kinase inhibitors (e.g., *p16*, *p21*, *p19*, and *p15*) were unchanged in LSK cells and HSCs from ME7-infected mice, compared to control mice (Supplementary Fig. S5B and C). Senescent HSCs can potentiate myeloid lineage-biased differentiation [25]. ME7-infected mice showed no bias toward myeloid-lineage (CD11b^+^) cells in the PB (Supplementary Fig. S5D).

In addition to cellular senescence, ROS can cause cell death in HSPCs [26]. BM cells from ME7-infected middle-aged mice showed significantly greater percentages of annexin V- and caspase 3-positive LSK cells and HSCs, compared to BM cells from control mice (Fig. 3C, D). Elevated ROS levels accompanied by MAPK activity can modulate diverse processes involved in the proliferation, differentiation, senescence, and death of stem cells [27, 28]. Compared with control mice, ME7-infected mice exhibited particularly extensive phosphorylation of JNK, but not extracellular signal-regulated kinase or p38, in LSK cells and HSCs (Fig. 3E). These results indicate that the ME7 infection-derived reduction of HSPC abundance is caused by infection-related induction of apoptotic cell death, rather than by regulation of the cell cycle or cellular senescence.

In contrast to ME7-infected middle-aged mice, ME7-infected old-aged mice, which were infected for 7 months, exhibited abnormal PB counts. The total number of WBCs, but not the numbers of RBCs and platelets, was significantly reduced in the PB of ME7-infected old-aged mice, compared to control mice (Supplementary Fig. S6A). ME7-infected old-aged mice exhibited a decrease in the proportion of circulating lymphocytes and an increase in that of granulocytes, compared to the corresponding control mice. Because of this decreased WBC abundance, significant decreases in the abundances of lymphocytes and granulocytes were observed in the PB of infected old-aged mice (Supplementary Fig. S6B). BM cellularity significantly decreased in ME7-infected old-aged mice, compared to the corresponding control mice (Supplementary Fig. S6C). These results suggest that ME7 infection modulates hematopoiesis by reducing WBC production during later stages of infection.

To determine whether the loss of HSPCs in ME7-infected middle-aged mice progresses with prolonged duration of infection, mice were kept until 7 months after ME7 infection, when the infected mice were near death (i.e., they exhibited weight loss and physical inactivity, symptoms of prion disease). Although the numbers of BM LSK cells and HSCs were significantly reduced in ME7-infected old-aged mice, compared to the corresponding control mice, ME7-infected old-aged mice did not exhibit progressive decreases in the numbers of LSK cells and HSCs compared to the same group of mice at 5 months post-infection (Supplementary Fig. S7A, total graphs on the right). These results led us to hypothesize that ME7 infection-induced cell death in HSPCs may be limited to cells that express PrP^C^. To test this hypothesis, we analyzed the numbers of PrP^C^-negative and PrP^C^-positive cells among LSK cells and HSCs from ME7-infected old-aged mice. As shown in Supplementary Fig. S7A, the numbers of PrP^C^-negative LSK cells and HSCs did not significantly differ between ME7-infected old-aged mice and the corresponding control mice. In contrast, the numbers of PrP^C^-positive LSK cells and HSCs significantly decreased, by 3.8-fold and 5.4-fold, respectively, in ME7-infected old-aged mice compared to control mice. Similarly, ME7-infected old-aged mice exhibited lower percentages of PrP^C^-positive LSK cells and HSCs, but not PrP^C^-negative cells, in a given total cell count. Our hypothesis was further supported by additional experiments that revealed increases in mitochondrial ROS level and annexin V-positive cells only in PrP^C^-positive LSK cells and HSCs, but not PrP^C^-negative cells, from ME7-infected middle-aged mice compared to the corresponding control mice (Supplementary Fig. S7B and C).

PrP^C^-null HSCs exhibit impaired self-renewal capacity with serial transplantation [16]. Transplantation experiments showed that ME7-infected LSK cells exhibited lower reconstitution capacity with serial transplantation, compared to control cells (Supplementary Fig. S7D). Overall, these results illustrate that ME7 infection causes a defect in HSPC functional integrity that is associated with ME7 infection-induced autonomous death of HSPCs expressing PrP^C^.

### ME7-infected mice exhibit HSC senescence during aging

**Fig. 4:**
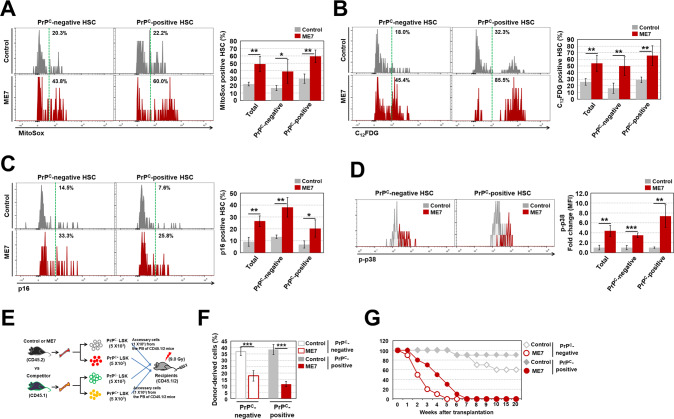
ME7-infected mice exhibit HSC senescence during aging. Levels of mitochondrial ROS (**A**) and SA-β-gal activity (**B**) in PrP^C^-negative and -positive BM HSCs from control and ME7-infected old-aged mice were measured (*n* = 7). Levels of p16 (**C**) and phospho-p38 (**D**) in PrP^C^-negative and -positive BM HSCs from both old-aged mouse groups were analyzed after cells had been fixed and permeabilized (*n* > 6). **E** For analysis of competitive repopulation capacity, PrP^C^-negative or PrP^C^-positive LSK cells from control or ME7-infected old-aged mice (CD45.2) were co-transplanted with equal numbers (5 × 10^3^) of PrP^C^-negative or PrP^C^-positive LSK cells from competitor mice (CD45.1) and accessory cells (1 × 10^6^) from the PB of non-conditioned recipient mice (CD45.1/2) into conditioned recipient mice (CD45.1/2, 900 rads). **F** The CD45.1/CD45.2 ratio in PB collected from recipient mice at 4 months post-transplantation was evaluated by flow cytometry (*n* = 5). **G** The survival rate was measured in recipient mice (CD45.1/2) that had been transplanted with CD45.2-expressing BM cells (5 × 10^5^) from primary recipient mice, which had been transplanted with LSK cells (*n* = 10). Donor cells were able to support recipient animal survival for 4–5 weeks because of short-term repopulating cells (i.e., radioprotective capacity); survival beyond 16 weeks requires long-term repopulating cells. All data are presented as means ± SDs. *p < 0.05, **p < 0.01, and ***p < 0.001 vs. control, as determined by Student’s *t* test.

Although ME7 infection specifically induced apoptotic cell death in PrP^C^-positive HSPCs, infection did not cause the death of all PrP^C^-positive HSPCs. Therefore, we further evaluated live PrP^C^-positive HSCs in ME7-infected old-aged mice. We first investigated whether HSCs from ME7-infected old-aged mice experience oxidative stress, as observed in infected middle-aged mice. The mitochondrial ROS level was continuously and significantly greater in HSCs from ME7-infected old-aged mice than in the corresponding control mice (Fig. 4A). Among HSCs from ME7-infected old-aged mice, PrP^C^-positive HSCs had a significantly increased level of mitochondrial ROS, compared to the corresponding control mice. Unexpectedly, enhanced ROS levels were also observed in PrP^C^-negative HSCs from ME7-infected old-aged mice (Fig. 4A). Notably, in contrast to ME7-infected middle-aged mice (Supplementary Fig. S5B), infected old-aged mice had greater SA-β-gal activity in HSCs than did the corresponding control mice. This ME7-induced increase in SA-β-gal activity of HSCs resulted from both PrP^C^-positive cells and PrP^C^-negative cells (Fig. 4B). In contrast to the results for SA-β-gal activity in ME7-infected old-aged mice, the percentages of annexin V-positive HSCs among PrP^C^-positive and PrP^C^-negative cells did not significantly change compared to control mice (Supplementary Fig. S8). Senescence factors such as p16 and p38, which are strongly expressed in senescent stem cells [29, 30], significantly increased in both PrP^C^-positive and PrP^C^-negative HSCs from ME7-infected old-aged mice, compared to control mice (Fig. 4C, D). These results suggest that cell death-tolerant ME7-infected HSCs undergo progressive senescence during aging; this process is not fully dependent on PrP^C^ expression.

Our hypothesis was further supported by competitive and non-competitive transplantation experiments (Fig. 4E and Supplementary Fig. S9). PrP^C^-positive LSK cells from ME7-infected old-aged mice exhibited lower repopulation capacity in conditioned recipient mice, compared to the same cells from control mice. Similarly, defective repopulation capacity was observed in transplanted ME7-infected PrP^C^-negative LSK cells (Fig. 4F). The defective repopulation capacity of ME7-infected senescent HSCs during competitive transplantation was further confirmed by an animal survival experiment. The results showed that mice transplanted with ME7-infected PrP^C^-positive or PrP^C^-negative BM cells began to die at 1-2 weeks after transplantation; all of these mice died within 7 weeks (Fig. 4G). These findings show that senescence occurrence in PrP^C^-negative HSCs, particularly from ME7-infected old-aged mice, can progress non-autonomously because of changes in the BM microenvironment; this process can negatively modulate HSCs.

### Preferential impairment of the BM microenvironment in ME7-infected mice leads to HSC senescence

As a stem cell niche, the BM microenvironment plays an important role in maintaining HSC functional integrity [31, 32]. Impairment of the BM microenvironment, particularly in relation to the aging phenotypes triggered by mesenchymal stem cells (MSCs), induces HSC senescence [33]. Based on our findings that ME7-infected mice undergo extensive adipogenesis (presumably because of senescent MSCs) in the BM (Supplementary Fig. S1D) [34], we sought to investigate the effect of ME7 infection on modulation of the MSC-mediated BM microenvironment. For this purpose, we first measured the expression of PrP^C^ in MSCs. Approximately 47.5% of BM-conserved MSCs expressed PrP^C^ protein (Supplementary Fig. S10A).

**Fig. 5:**
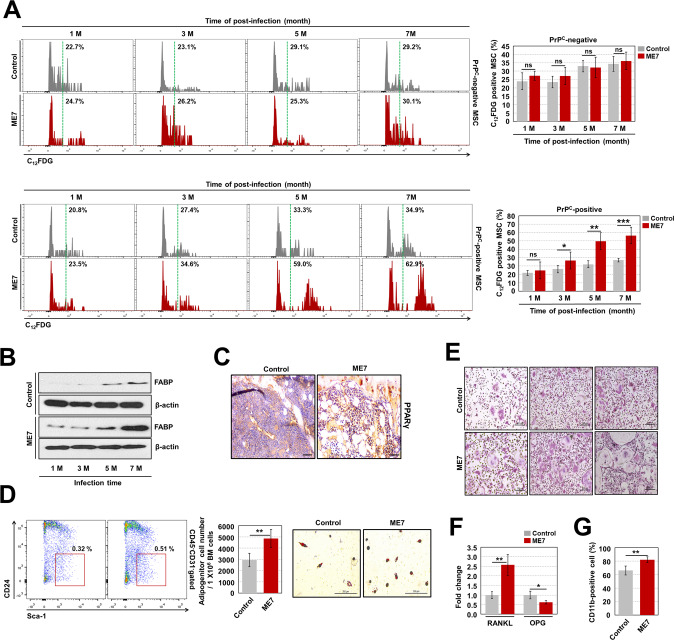
ME7 infection causes the impairment of BM microenvironment with aging phenotypes. **A** SA-β-gal activities in PrP^C^-negative and -positive BM MSCs (phenotypically defined as Lin^-^Sca-1^+^CD29^+^CD105^+^ cells) from control and ME7-infected mice were measured at the indicated times after infection (*n* = 6). **B** The expression levels of fatty acid-binding protein (FABP) in BM cells derived from control and ME7-infected mice were measured by western blotting. **C** The intensity of peroxisome proliferator activated receptor gamma (PPARγ) protein in the BM of ME7-infected old-aged mice was measured by immunohistochemistry. Scale bars are 200 µm. A representative result is shown (*n* = 5). **D** The numbers of adipogenic progenitors (phenotypically defined as CD45^-^CD31^-^Sca-1^+^CD24^−^ cells) in the BM of control and ME7-infected old-aged mice were assessed by flow cytometry (*n* = 5), while adipogenesis was measured by oil red O staining after BM cells had been incubated in adipogenic differentiation medium, as described in the Materials and Methods. Scale bars are 200 µm. Representative data from three independent experiments are shown. **E** For analysis of osteoclast activity, BM cells were incubated for 5 days in osteoclast differentiation medium, as described in the Materials and Methods, then stained using a TRAP assay. Scale bars are 200 µm. Representative data from three independent experiments are shown. **F** Levels of RANKL and osteoprotegerin (OPG) were measured in cell-free BM supernatant from old-aged mice using a sandwich enzyme-linked immunosorbent assay kit; representative data from three independent experiments are shown. **G** Proportions of monocytes and macrophages in the BM of old-aged mice were assessed by incubating the cells with CD11b antibody (*n* = 6). All data are presented as means ± SDs. *p < 0.05, **p < 0.01, and ***p < 0.001 vs. control, as determined by Student’s *t* test.

ME7-infected mice exhibited no change in the number of MSCs during the infection period, compared to control mice (Supplementary Fig. S10B). These results were supported by comparable percentages of annexin V-positive MSCs in both groups (Supplementary Fig. S10C). However, SA-β-gal activity was significantly increased in MSCs from ME7-infected mice, compared to control mice. The significant increase in SA-β-gal activity was limited to ME7-infected PrP^C^-positive MSCs and began at 3 months post-infection; however, it was not observed in ME7-infected PrP^C^-negative MSCs (Fig. 5A).

The aged BM microenvironment exhibits a tendency toward increased adipogenesis and osteoclastogenesis [35, 36]. As shown in Supplementary Fig. S1D, extensive adipogenesis was observed in the BM of ME7-infected old-aged mice. Cells of the infected mice had high expression levels of adipogenesis-associated markers such as FABP and PPARγ in the BM of ME7-infected old-aged mice (Fig. 5B, C). This result was associated with an increase in the number of adipogenic progenitors in the infected BM and an increase in the number of oil red O-stained adipocytes (Fig. 5D).

Adipogenesis in aged BM is linked to extensive osteoclast activity, accompanied by increased expression of RANKL. Similarly, BM cells from ME7-infected old-aged mice had substantially greater numbers of TRAP-stained multinuclear osteoclasts than did BM cells from the corresponding control mice (Fig. 5E). These results were supported by the significantly increased level of RANKL, decreased level of osteoprotegerin, and greater number of CD11b^+^ macrophages in the BM of ME7-infected old-aged mice, compared to control mice (Fig. 5F, G). However, no differences in the levels of osteogenesis-related factors (e.g., Runx2, osterix, and osteopontin), bone mineralization capacity and femoral bone parameter were observed between ME7-infected old-aged mice and the corresponding control mice (Supplementary Fig. S11A–C). These findings suggest that ME7 infection leads to impairment and aging of the BM microenvironment.

**Fig. 6:**
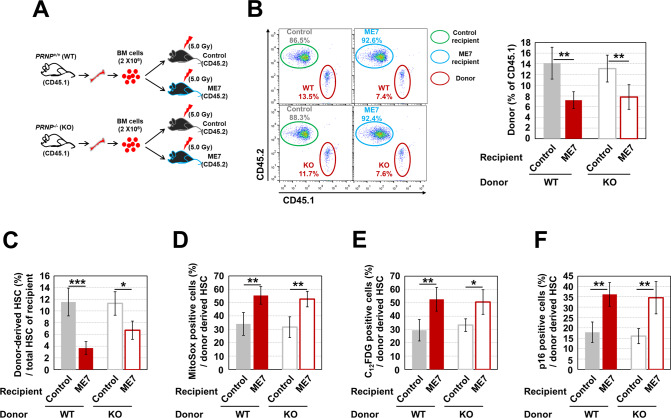
ME7 infection-related impairment of BM renders HSCs senescent. **A** Schematic diagram of the transplantation experiment to determine whether HSC senescence in ME7-infected mice was due to ME7-mediated preferential impairment of the BM microenvironment. Normal BM cells (2 × 10^6^, CD45.1) from *Prnp*^+/+^ (WT) or *Prnp*^−/−^ (KO) mice were non-competitively transplanted into control or ME7-infected mice that had received sub-lethal irradiation at 3 months post-infection (CD45.2, 500 rads, *n* = 5). **B** The CD45.1/CD45.2 ratio in PB of recipient mice at 4 months post-transplantation was evaluated by flow cytometry (*n* = 5). Percentage (**C**, *n* = 5), mitochondrial ROS level (**D**, *n* = 5), SA-β-gal activity (**E**, *n* = 5), and p16 expression level (**F**, *n* = 5) of donor cell-derived HSCs were analyzed in the BM of transplanted recipient mice. All data are presented as means ± SDs. *p < 0.05, **p < 0.01, and ***p < 0.001 vs. control, as determined by Student’s *t* test.

To better understand whether HSC senescence in ME7-infected mice is progressively caused by impairment of the BM microenvironment, we performed a non-competitive transplantation experiment that involved transplanting BM cells (2 × 10^6^, CD45.1) from *Prnp*^+/+^ (WT) or *Prnp*^−/−^ (KO) mice, as confirmed by PCR (Supplementary Fig. S12A), into control or ME7-infected mice that had received sub-lethal irradiation at 3 months post-infection (CD45.2, 5 Gy, Fig. 6A). We confirmed the expression level of PrP^C^ in BM-conserved hematopoietic cells from KO mice that had been selected via genotyping analysis (Supplementary Fig. S12B).

The repopulating percentage of WT-derived donor cells significantly decreased in the PB of ME7-infected recipient mice, compared to non-infected recipient mice, at 16 weeks post-transplantation (Fig. 6B). Similarly, a decrease in the repopulating percentage of KO-derived donor cells was observed in the PB of infected recipient mice (Fig. 6B). The BM of conditioned ME7-infected recipient mice exhibited significantly reductions in the percentages of WT- and KO-derived HSCs, compared to the corresponding non-infected recipient mice (Fig. 6C). WT- and KO-derived HSCs transplanted into conditioned ME7-infected recipient mice exhibited substantially higher levels of senescence-associated markers (e.g., mitochondrial ROS, SA-β-gal activity, and p16 protein) than did the same cells transplanted into non-infected recipient mice (Fig. 6D–F). These findings suggest that the BM of ME7-infected mice does not provide an optimal environment for the engraftment and maintenance of HSCs, which do not depend on PrP^C^.

### ME7-mediated modulation of HSCs and the BM microenvironment is dependent on PrP^C^-based signal induction

To investigate whether ME7 infection-induced phenomena in HSCs and the BM microenvironment are dependent on PrP^C^, we injected ME7 into *Prnp* WT and KO mice. Weight loss and physical inactivity were observed in ME7-infected old-aged WT mice, but not in the corresponding infected KO mice (Supplementary Fig. S13A and Supplementary Video S2). In addition, no PrP^C^ or PrP^Sc^ bands appeared in samples that were derived from the brains of ME7-infected KO mice at 5 and 7 months post-infection (Supplementary Fig. S13B and C). Mitochondrial ROS levels significantly increased in HSCs from ME7-infected WT mice, but not in those from the infected KO mice (Supplementary Fig. S14A). Apoptotic cell death-related factors such as annexin V and caspase 3 increased in HSCs from ME7-infected middle-aged WT mice, but not infected old-aged WT mice, compared to HSCs from non-infected middle-aged WT mice. In contrast, those factors were not increased in HSCs from ME7-infected middle- and old-aged KO mice, compared to non-infected mice of the same ages (Supplementary Fig. S14B and C). Senescence-related factors (e.g., SA-β-gal activity and p16 protein expression) were elevated in HSCs from ME7-infected old-aged WT mice but not in HSCs from infected middle-aged WT mice. Conversely, comparable levels of senescent factors were observed in HSCs from ME7-infected and non-infected KO mice (both middle-aged and old-aged) (Supplementary Fig. S14D and E). The phosphorylation levels of JNK and p38 tended to increase in ME7-infected middle-aged and old-aged WT mice, respectively. However, MAPK levels were unchanged in HSCs from ME7-infected middle-aged and old-aged KO mice (Supplementary Fig. S14F and G). A decrease in the number of colonies was observed in ME7-infected middle-aged WT mice, but not in infected middle-aged KO mice (Supplementary Fig. S14H). Similarly, no changes in the numbers of WBCs, RBCs, or platelets were observed in the PB of ME7-infected middle- and old-aged KO mice, compared to the corresponding non-infected KO mice (Supplementary Fig. S15).

SA-β-gal activities were significantly greater in MSCs from ME7-infected middle- and old-aged WT mice-but did not change in MSCs from infected KO mice-compared to non-infected WT and KO mice, respectively, of the same ages (Supplementary Fig. S14I). In addition, the intensities of adipogenesis and osteoclastogenesis were increased in the BM of ME7-infected middle- and old-aged WT mice, compared to the corresponding non-infected KO mice (Supplementary Fig. S14J-M). Taken together, these findings demonstrate that ME7-targeted PrP^C^ triggers signaling pathways capable of modulating HSC fate through both cell-autonomous and non-autonomous mechanisms.

## Discussion

Similar to previous findings that various types of hematopoietic cells express PrP^C^ at differing levels [14–17], the present results indicate that hematopoietic cells express PrP^C^ protein at different levels depending on the stage of cell maturation; in particular, the number of PrP^C^-positive cells is greater in primitive HSCs than in other mature hematopoietic cells (Fig. 1A). This prion protein reportedly has an important role in the maintenance of self-renewal capacity for long-term repopulation of HSCs [17]. In addition, PrP^C^ can protect HPCs against genotoxic stress [37]. However, the mechanism that underlies the modulation of HSPC fate and hematopoiesis through the conformational alteration of PrP^C^ to PrP^Sc^ has remained unclear. To investigate this mechanism, we infected C57BL/6 mice with prion strain ME7 through i.p. injection; we confirmed the presence of the conformationally altered isoform of PrP^Sc^ in the brain and BM of the infected mice. Our results provide important insights regarding the effect of ME7-induced PrP^Sc^ formation on BM-conserved HSPCs.

Prion-infected brains develop oxidative stress-induced mitochondrial dysfunction, followed by prion disease progression and neuronal cell death [38, 39]. Similarly, we found that PrP^C^-positive HSPCs, but not PrP^C^-negative HSPCs (i.e., from *Prnp* KO mice), from prion-infected mice exhibit increased levels of mitochondrial superoxide anion (Fig. 3A, D, and Supplementary Fig. S14A). To our knowledge, this is the first report that prion-infected PrP^C^-positive HSPCs exhibit cell-autonomous apoptotic cell death due to ME7-induced upregulation of mitochondrial ROS, JNK phosphorylation, and caspase 3 expression (Fig. 3A–E, Supplementary Figs. S7A–C, and S14A–C and F).

MSCs can modulate the balance of osteogenesis and adipogenesis in the BM [40]. MSCs undergoing senescence can become biased toward adipogenesis [35]. The present study is the first report of PrP^C^ protein expression in MSCs (Supplementary Fig. S10A). In contrast to infected PrP^C^-positive HSPCs, prion-infected PrP^C^-positive MSCs undergo cellular senescence, which is characterized by enhanced SA-β-gal activity and increased differentiation toward adipogenesis; however, they do not undergo cell death (Fig. 4A–D and Supplementary Fig. S10C). Thus, the BM of prion-infected mice exhibits severe adipogenesis without obvious changes in osteogenesis (Supplementary Fig. S11). The present findings regarding distinct modulation processes in prion-infected PrP^C^-positive HSPC and MSC fate suggest that prion-targeted PrP^C^ may trigger the induction of cell type-specific signaling processes related to cell-fate determination.

The BM microenvironment is essential for maintaining lifelong homeostasis of HSC functional integrity [31, 32]. In addition to a bias toward adipogenesis, the aged BM microenvironment exhibits extensive osteoclastogenesis and non-autonomous progression of HSC senescence [33]. Similarly, prion-infected BM exhibits robust adipogenesis and enhanced osteoclastogenesis; these processes eventually render both PrP^C^-positive and negative HSCs senescent in a non-cell autonomous manner, as revealed by our non-competitive transplantation experiment (Fig. 6A–F).

Based on our current findings on prion infection and studies on the role of PrP^C^ in the modulation of HSPCs, studies on the functional difference between PrP^C^-positive and -negative HSCs under physiological and stressful conditions need to be further conducted for a greater understanding of the functional role of subpopulations of HSCs depending on PrP^C^ expression.

**Fig. 7:**
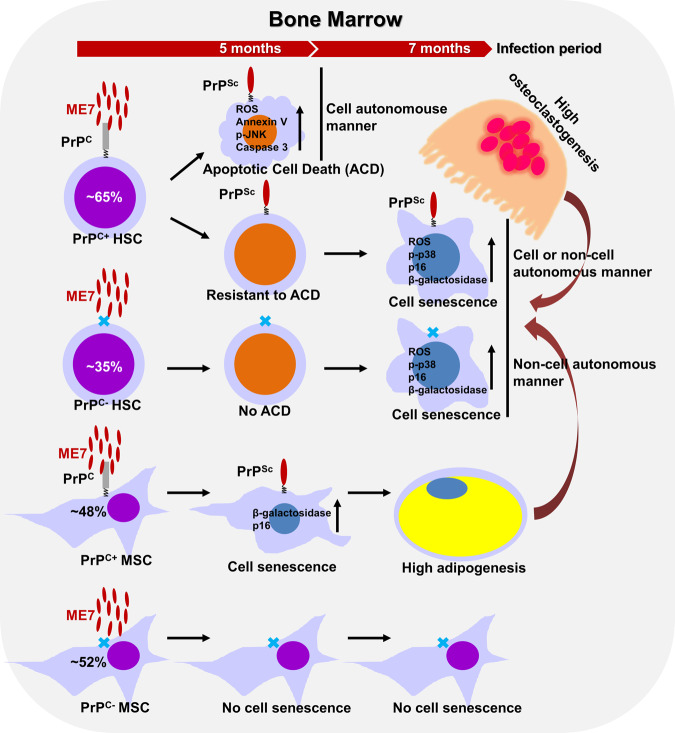
A simplified schema of the proposed model. PrP^C^-positive HSCs, but not PrP^C^-negative ones, in ME7-infected middle-aged mice at 5 months post-injection undergo an apoptotic cell death through a cell autonomous manner. Both of PrP^C^-negative and positive HSCs in ME7-infected old-aged mice at 7 months post-injection exhibit senescence by the preferential aged BM microenvironment through a non-cell autonomous manner.

In conclusion, our findings reveal a new avenue for investigations of prion disease, whereby the disease is accompanied by hematopoietic defects that lead to apoptotic cell death and senescence of HSCs through both autonomous and non-autonomous mechanisms (Fig. 7). The discovery of hematopoietic defects in prion disease offers the potential for improved survival in patients with prion disease; it also suggests a need for caution regarding the clinical use of BM cells from early prion-infected individuals without prion disease-related symptoms for transplantation in cancer patients.

## Supplementary information

Supplementary Information_clean version

Supplementary Figures

Video S1A

Video S1B

Video S1C

Video S1D

Video S2A

Video S2B

Video S2C

Video S2D

Video S2E

Video S2F

Video S2G

Video S2H

## Data Availability

Data supporting the presented findings may be shared upon request.
