# Supplementary material for: Prion infection modulates hematopoietic stem/progenitor cell fate through cell-autonomous and non-autonomous mechanisms
Source: Leukemia. 2023 Jan 27;37(4):877–87. doi: 10.1038/s41375-023-01828-w (PMC10079512; doi:10.1038/s41375-023-01828-w)
Supplement: Supplementary file 1 — Supplementary Information_clean version [file 41375_2023_1828_MOESM1_ESM.docx]

**Prion infection modulates hematopoietic stem/progenitor cell fate through cell-autonomous and non-autonomous mechanisms**

Hyun-Jaung Sim^1,2#^, Yong-Chan Kim^1,3#^, Govinda Bhattarai^1,2#^, Sae-Young Won^1,3^, Jeong-Chae Lee^1,2*^, Byung-Hoon Jeong^1,3*^, Sung-Ho Kook^1,2*^

^1^Department of Bioactive Material Sciences, Research Center of Bioactive Materials, Jeonbuk National University, Jeonju 54896, Republic of Korea

^2^Cluster for Craniofacial Development and Regeneration Research, Institute of Oral Biosciences and School of Dentistry, Jeonbuk National University, Jeonju 54896, Republic of Korea

^3^Korea Zoonosis Research Institute, Jeonbuk National University, Iksan 54531, Republic of Korea

^*^Address correspondence to: Jeong-Chae Lee, Institute of Oral Biosciences and School of Dentistry, Jeonbuk National University, Jeonju 54896, Republic of Korea, Phone: +82-63-270-4049, Fax: +82-63-270-4004, E-mail: [jeongchae@jbnu.ac.kr](mailto:jeongchae@jbnu.ac.kr)

Byung-Hoon Jeong, Korea Zoonosis Research Institute, Jeonbuk National University, Jeonbuk, 54531, Republic of Korea, Phone: +82-63-900-4040, Fax: +82-63-900-4012, E-mail: bhjeong@jbnu.ac.kr

Sung-Ho Kook, Department of Bioactive Material Sciences, Jeonbuk National University, Jeonju 54896, Republic of Korea, Phone: +82-63-270-3327, Fax: +82-63-270-4312, E-mail: [kooksh@jbnu.ac.kr](mailto:kooksh@jbnu.ac.kr)

^#^These authors contributed equally to this work

**Supplementary Information**

**Materials and Methods**

**Genotyping of *Prnp* knockout mice**

The polymerase chain reaction (PCR) mixture comprised 2.5 µL of 10× H-star *Taq* reaction buffer, 5 µL of 5× band helper, 1 µL of 10 mM dNTP mix, 1 µL of each primer (10 µM), 0.2 µL of H-star *Taq* DNA polymerase (BIOFACT, Daejeon, Korea), and nuclease-free water to a total volume of 25 µL. The PCR conditions were as follows: denaturation at 95°C for 15 min; 34 cycles of 95°C for 20 s, 56°C for 40 s, and 72°C for 1 min for annealing and extension; and final extension at 72°C for 5 min. PCR was performed using a C1000 Touch Thermal Cycler (Bio-Rad, Hercules, CA, USA). The PCR product was stained with ethidium bromide, separated via electrophoresis in a 1% agarose gel, and assessed for genotyping (wild-type allele: 388 bp; mutant allele: 341 bp). The PCR products were sequenced on an ABI 3730 sequencer (Applied Biosystems, Foster City, CA, USA) and the DNA sequences of the amplicons were confirmed using Finch TV software (Geospiza Inc., Seattle, WA, USA).

**Evaluation of proteinase K-resistant fibrils via western blotting analysis**

Brain and BM cell samples were collected and homogenized with 10% volumes of Radioimmunoprecipitation assay (RIPA) Lysis Buffer (Thermo Fisher Scientific, USA) containing a complete protease inhibitor cocktail (Roche, Germany). The homogenates were centrifuged at 14,000 rpm for 15 min at 4°C. Protein concentrations were estimated via bicinchoninic acid assays (Bio-Rad). To detect the PrP^Sc^ band, samples were incubated with 40 µg/mL of proteinase K for 1 h at 37°C; they were then heated to 95°C for 10 min and separated by electrophoresis in a 12% sodium dodecyl sulfate gel. The separated proteins were transferred to a nitrocellulose membrane (Amersham, USA) using an electrophoretic transfer system (Bio-Rad) at 100 V for 90 min. The membranes were washed with a Tris-buffered saline solution (pH 7.6) containing 0.05% Tween 20 (TBST), then blocked in TBST containing 5% skim milk (Santa Cruz Biotechnology, USA) for 90 min at room temperature. Subsequently, membranes were incubated at 4°C overnight with mouse monoclonal anti-PrP antibody (SAF84, 1:200). After an additional wash in TBST, the membranes were incubated with horseradish peroxidase-conjugated anti-mouse immunoglobulin G antibody (IgG) (Sigma-Aldrich, USA) for 1 h at room temperature, then washed again in TBST. The target signals were detected using Pierce ECL Western Blotting Substrate (Thermo Fisher Scientific).

**Flow cytometry**

The numbers of BM cells collected from mice that had been sacrificed via CO_2_ asphyxiation were measured by multi-color flow cytometry (BD Aria; BD Biosciences, Franklin Lakes, NJ, USA) at the Center for University-Wide Research Facilities of Jeonbuk National University. The populations of hematopoietic cells from the BM were phenotypically gated, then analyzed using FlowJo software (FLOWJO; Ashland, OR, USA). Anti-PrP^C^ antibody (SPI Bio) was biotinylated using the EZ-Link Micro N-hydroxysuccinimide ester (NHS)-polyethylene glycol (PEG4)-Biotinylation Kit (Pierce), in accordance with the manufacturer’s instructions. Lin^-^Sca-1^+^c-Kit^+^ (LSK) cells and HSCs were phenotypically characterized using the following antibodies (purchased from BD Biosciences unless otherwise specified): lineage markers phycoerythrin (PE)-Cy7-conjugated anti-CD3, anti-CD4, anti-CD8, anti-CD45R, anti-CD11b, anti-Gr-1, and anti-TER-119; PE-conjugated or fluorescein isothiocyanate (FITC)-conjugated anti-Sca-1; allophycocyanin-conjugated anti-c-Kit; PerCP/Cy5.5-conjugated anti-CD150 (eBioscience); allophycocyanin–Cy7-conjugated anti-CD48. BM-derived HPCs (e.g., granulocyte-monocyte progenitors, common myeloid progenitors, megakaryocyte-erythroid progenitors, and common lymphoid progenitors) were characterized using PE-conjugated anti-FcR and PerCP/Cy5.5-conjugated anti-CD34 (BioLegend) on the basis of Lin^-^Sca-1^-^c-Kit^+^ markers, as well as PE-conjugated anti-IL-7R. The proportions of circulating monocytes (CD11b^+^) and B-cells (B220^+^) in the peripheral blood (PB) were measured using FITC- and PE-conjugated antibodies, respectively. The mitochondrial superoxide anion level and senescence-associated beta-galactosidase (SA-β-gal) activity were measured with MitoSox Red (Invitrogen) and 5-dodecanoylaminofluorescein di-β-D-galactopyranoside (C_12_FDG; Molecular Probes), respectively. Apoptotic cell death was identified with an annexin V detection kit (BD Biosciences) and caspase 3 (BD Biosciences). The protein levels of Ki-67 (BD Biosciences) and Nrf2 (Cell Signaling) were measured with FITC-conjugated antibodies after cells had been fixed and permeabilized. Mitogen-activated protein kinase (MAPK) phosphorylation was assessed using Alexa Fluor 488-conjugated anti-p38 MAPK (BD Biosciences), PE-conjugated anti-c-Jun N-terminal kinase (JNK; BD Biosciences) and Alexa Fluor 488-conjugated anti-extracellular signal-regulated kinase (BD Biosciences). The level of p16 protein expression (Santa Cruz Biotechnology) was measured using FITC-conjugated antibody after cells had been fixed and permeabilized.

**Transplantation experiment**

To evaluate donor cell-derived reconstitution capacity, recipient mice were lethally irradiated (9 Gy) 8–24 h prior to transplantation. BM cells (5 × 10^5^) and LSK cells (5 × 10^3^) from control or ME7-infected mice (CD45.2) were co-transplanted with equal numbers of the same cells from competitor mice (CD45.1) into conditioned recipient mice (CD45.2) via tail vein injection. To measure stem cell self-renewal capacity, BM cells (1 × 10^6^) from the initial recipient mice were serially transplanted into conditioned recipient mice. The donor cell-mediated reconstitution capacity in the PB of recipient mice was assessed at the indicated times after transplantation. The engraftment capacity of donor-derived HSPCs was measured in the BM of tertiary recipient mice. For analysis of competitive reconstitution capacity depending on PrP^C^ expression, equal numbers (5 × 10^3^) of PrP^C^-negative or positive LSK cells that had been collected from control mice or mice (CD45.2) at 7 months after ME7 infection (hereafter referred to as ME7-infected old-aged mice) were co-transplanted with PrP^C^-negative or PrP^C^-positive LSK cells from competitor mice (CD45.1) and accessory cells (1 × 10^6^) from the PB of non-conditioned recipient mice (CD45.1/2) into conditioned recipient mice (CD45.1/2, 900 rads). To determine the capacity of donor cells to support the survival of the recipient mice, CD45.2-expressing BM cells (5 × 10^5^) from the primary recipient mice were non-competitively transplanted into conditioned recipient mice (CD45.1/2). To determine the fate of donor cells in host mice, normal BM cells (2 × 10^6^, CD45.1) from wild-type (WT) or knockout (KO) mice were non-competitively transplanted into control or ME7-infected mice that had received sub-lethal irradiation at 3 months post-infection (CD45.2, 5 Gy). PB cells were collected from host mice at 4 months post-transplantation, and the ratio of CD45.1/CD45.2 was measured. The percentage, mitochondrial reactive oxygen species (ROS) level, SA-β-gal activity, and p16 expression level of donor cell-derived HSCs were analyzed in the BM of recipient mice at 4 months post-transplantation.

**Colony-forming unit (CFU) assay**

To assess HSPC activity, BM cells (2 × 10^4^ per dish) were seeded into 35-mm dishes with MethoCult GF M3434 (Stem Cell Technologies) for CFU assays. After incubation for 12 days, the numbers of CFUs of granulocyte/macrophage (CFU-GM) and granulocyte/erythrocyte/macrophage/megakaryocyte (CFU-GEMM) cells and burst-forming units of erythrocytes (BFU-E) were counted using standard criteria. To measure clonogenic formation of pre-B progenitors, BM cells (2 × 10^5^ per dish) were seeded in 35-mm dishes with MethoCult M3630 (Stem Cell Technologies) and the numbers of colonies were counted after 7 days of incubation.

**Blood counts**

PB samples from control and ME7-infected mice were collected in Vacutainer (BD Biosciences) plastic tubes coated with K_2_-ethylenediaminetetraacetic acid. The numbers of white blood cells (WBCs), lymphocytes, granulocytes, red blood cells (RBCs) and platelets, as well as the percentages of lymphocytes and granulocytes, were measured with an automated complete cell counter (PE-6800VET; Prokan).

**Section and staining of femoral bone**

Femoral bones from each group of mice were fixed in 4% paraformaldehyde solution for 24 h, then decalcified in 10% ethylenediaminetetraacetic acid at 4°C for 6 weeks. The decalcified specimens were dehydrated, embedded in paraffin, and sliced into 5.0-µm parasagittal sections. For hematoxylin and eosin staining, the sections were de-paraffinized and rehydrated; they were then treated with Gill No. 3 hematoxylin (Sigma-Aldrich) and counterstained with 0.25% Eosin Y (Sigma-Aldrich). Subsequently, tissue sections were subjected to tartrate-resistant acid phosphatase (TRAP) staining using a leukocyte acid phosphatase kit (Cosmo Bio, Tokyo, Japan), then counterstained with hematoxylin. Some tissue sections were subjected to immunohistochemistry to determine the levels of prion PrP^Sc^ (HycultBiotech), adiponectin (Abcam), receptor activator of nuclear factor kappa-Β ligand (RANKL; BioWorld), and peroxisome proliferator-activated receptor gamma (PPARу; Santa Cruz Biotechnology), using an immunohistochemistry accessory kit (Vector Laboratories, Burlingame, CA, USA). Each antibody was used at a dilution of approximately 1:200–400. All TRAP and immunohistochemistry staining procedures were performed in accordance with the appropriate manufacturer’s instructions. Stained tissue samples were observed and photographed using the MoticEasyScan system.

**Osteoclast differentiation and *in vitro* TRAP staining**

For osteoclast differentiation, BM cells were isolated from control or ME7-infected (7-month) mice; they were seeded into 12-well culture plates at a density of 10,000 cells per well, in the presence of 30 ng/mL murine macrophage colony-stimulating factor and 50 ng/mL murine RANKL (PeproTech, USA). Fresh culture medium was provided on day 3. After 5 days of incubation, the cultures were fixed with 4% paraformaldehyde in phosphate-buffered saline, then stained with the TRAP Staining Kit (Cosmo Bio, Tokyo, Japan), in accordance with the manufacturer’s instructions. TRAP-positive cells were counted via light microscopy; cells that contained ≥ 3 nuclei were considered osteoclasts.

**Bone mineralization assay**

BM cells from control and ME7-infected mice were incubated for 21 days in the presence of osteogenic medium (α-minimum essential medium containing 5% fetal bovine serum, 50 μM ascorbic acid, 100 nM dexamethasone, and 10 mM β-glycerophosphate). Alizarin red staining was performed to determine the degree of mineralization. In brief, cells were fixed for 20 min in 70% cold ethanol, then washed three times with ice-cold phosphate-buffered saline. The cells were stained for 5 min with alizarin red and observed under a light microscope.

**Adipocyte differentiation and oil red O staining**

BM cells from control and ME7-infected mice were incubated in adipogenic differentiation medium. Briefly, cells were seeded in 12-well culture plates with α-minimum essential medium containing 5% fetal bovine serum; incubated with dexamethasone (500 nM), 3-isobutyl-l-methylxanthine (500 µM), indomethacin (100 µM), and insulin (10 µg/mL) for 72 h to induce adipocyte differentiation; and then cultured with 10 µg/mL insulin for 14 days to maintain adipocyte differentiation. Lipid accumulation in BM cells was detected via oil red O staining. Cells were rinsed with phosphate-buffered saline and fixed in 10% formaldehyde solution for 1 h. The fixed cells were washed with distilled water, then stained with oil red O solution (Sigma-Aldrich) for 15 min.

**Western blot analysis**

Briefly, whole protein lysates from the BM of either the control or ME7-infected mice and protein extracts (20 µg per sample) were separated via sodium dodecyl sulfate-polyacrylamide gel electrophoresis on 10% gels, then electroblotted onto polyvinylidene difluoride membranes. The resulting blots were washed with a buffer containing 10 mM Tris-HCl (pH 7.6), 150 mM NaCl, and 0.05% Tween 20; they were then blocked in 5% skim milk for 1 h prior to incubation with primary antibodies. The membranes were washed, then incubated with horseradish peroxidase-conjugated goat anti-rabbit IgG or goat anti-mouse IgG. Immunoreactive bands were visualized with an enhanced peroxidase detection kit (ELPIS-Biotech, Taejeon, Korea) and imaged by exposure to X-ray film (Eastman Kodak, Rochester, NY, USA). Western blot analyses were conducted using antibodies specific for the following proteins: runt-related transcription factor 2 (Runx2; BS2831; BioWorld), osterix (Abcam), osteopontin (Abcam), and fatty acid-binding protein (Santa Cruz Biotechnology). An anti-β-actin antibody (Santa Cruz Biotechnology) was used as an internal control.

**Micro-computed tomography analysis**

Hind limbs from control and ME7-infected mice were scanned using a desktop scanner (1076 Skyscan Micro-CT; Skyscan, Kontich, Belgium) and analyzed with CTAn software (Skyscan).

**Real-time reverse transcription-PCR**

Total RNA was extracted with TRIzol reagent (Invitrogen Corp., Carlsbad, CA, USA), in accordance with the manufacturer’s instructions. RNA samples (1 µg per reaction) from individual mice were used to prepare cDNA for real-time reverse transcription-PCR with the AmpiGene^TM^ cDNA Synthesis Kit (Enzo Life Sciences, Farmingdale, NY, USA). Power SYBR Green PCR Master Mix (Life Technologies, CA, USA) was used to detect the accumulation of PCR products. The reaction was conducted using the ABI StepOnePLUS sequence detection system (Applied Biosystems) with the following thermal cycling program: pre-denaturation at 95°C for 2 min, followed by 40 cycles of denaturation at 95°C for 5 s, annealing at 60°C for 30 s, and extension at 65°C for 30 s. Primer sequences specific to *P15*, *P16*, *P19* and *P21* were used, as shown in Supplementary Table 1. *GAPDH* was used as an endogenous reference gene for quantification.

**Figure Legends**

**Fig. S1 Biotinylation of PrPC antibody and generation of the mouse model of prion disease.**

**A** PrP^C^ antibody was incubated with N-hydroxysuccinimide ester (NHS)-polyethylene glycol (PEG4)-biotin solution on ice for 2 h. The PrP^C^ antibody solution was then placed in the center of a Zeba Desalt Spin Column until absorption into the resin of the column; this was followed by centrifugation at 1,000 × g for 2 min to obtain biotin-labeled PrP^C^ antibody, in accordance with the manufacturer’s instructions. The specificity of the biotinylated antibody was analyzed using BM cells. **B** Generation of the mouse model of prion disease through i.p. injection of the ME7 scrapie strain and measurement of ME7-infected mouse body weights at 3, 5, and 7 months post-injection (n = 15). **C** Western blot bands of PrP^Sc^ were evaluated in homogenates of brain tissue and BM cells from control and ME7-infected mice at 3, 5, and 7 months post-injection (n = 5) via western blotting analysis using proteinase K, which can fully digest PrP^C^ protein but not PrP^Sc^ protein; a representative result is shown. Upper gel shows PrP^Sc^ bands from homogenates of mouse brain tissue. Lower gel shows PrP^Sc^ bands from homogenates of mouse BM cells. **D** PrP^Sc^ expression was measured in the BM of ME7-infected mice via immunohistochemistry (n = 5); a representative result is shown. Scale bars are 200 µm. All data are presented as means ± SDs. ***p < 0.001 vs. control, as determined by Student’s *t*-test.

**Fig. S2 Bioassay of prion infectivity using hematopoiesis-related cells such as HPCs and HSCs derived from ME7-infected mice.**

**A** Bioassay results from first-passage mice at 5 months post-injection. The first panel shows western blot bands of PrP^Sc^ in homogenates of brain tissue from mice that had been inoculated with HPCs from prion-infected mice (n = 5). The second panel shows western blot bands of PrP^Sc^ in homogenates of brain tissue from mice that had been inoculated with HSCs from prion-infected mice (n = 4). **B** Bioassay results of second-passage mice at 5 months post-injection. The first panel shows western blot bands of PrP^Sc^ in homogenates of brain tissue from second-passage mice that had been inoculated with homogenates of brain tissue from first-passage mice, which had been inoculated with HPCs from prion-infected mice (n = 5). The second panel shows western blot bands of PrP^Sc^ in homogenates of brain tissue from second-passage mice that had been inoculated with homogenates of brain tissue from first-passage mice, which had been inoculated with HSCs from prion-infected mice (n = 5).

**Fig. S3 Hematopoiesis in ME7-infected middle-aged mice.**

**A** The numbers of white blood cells (WBCs), red blood cells (RBCs), and platelets in the PB of ME7-infected middle-aged mice were assessed using an automated complete cell counter (n = 6). **B** The proportions and numbers of lymphocytes and granulocytes were analyzed among circulating leukocytes of the infected middle-aged mice (n = 6). **C** Total BM cellularity in the infected middle-aged mice was measured by trypan blue staining (n = 5). All data are presented as means ± SDs. ***p < 0.001 vs. control, as determined by Student’s *t*-test.

**Fig. S4** **ME7-infected middle-aged mice exhibit reduced HSPC abundance in the BM.**

**A** The numbers of BM-conserved HPCs, LSK cells, and HSCs, which characterized the phenotype described in Fig. 1A, were determined in control and ME7-infected middle-aged mice (n = 7). **B** and **C** The frequencies of HPCs, which were further classified using anti-FcR and anti-CD34 antibodies as Lin^-^Sca-1^-^c-Kit^+^ markers, along with an IL-7R antibody (n = 7, GMP, granulocyte-monocyte progenitor; CMP, common myeloid progenitor; MEP, megakaryocyte-erythroid progenitor; CLP, common lymphoid progenitor) were measured. All data are presented as means ± SDs. **p < 0.01 and ***p < 0.001 vs. control, as determined by Student’s *t*-test.

**Fig. S5 ME7 infection does not modulate the cell cycle or senescence in HSPCs.**

**A** The proliferation of LSK cells and HSCs in the BM of control and ME7-infected middle-aged mice was analyzed using an anti-Ki-67 antibody (n = 7). **B** SA-β-gal activities in BM LSK cells and HSCs from both mouse groups were analyzed by incubating the cells with C_12_FDG, a β-galactosidase substrate (n = 7). **C** mRNA levels of *p15, p16, p19,* and *p21* were measured in LSK cells from the BM of both mouse groups; representative results from three independent experiments are shown. **D** Circulating lymphoid (B220^+^) and myeloid (CD11b^+^) lineages were measured in the PB of both mouse groups (n = 7).

**Fig. S6 Hematopoiesis in ME7-infected old-aged mice.**

**A** The numbers of WBCs, RBCs, and platelets in the PB of ME7-infected old-aged mice were assessed using an automated complete cell counter (n = 7). **B** The proportions and numbers of lymphocytes and granulocytes were analyzed among circulating leukocytes of the infected old-aged mice (n = 7). **C** Total BM cellularity in the infected old-aged mice was measured by trypan blue staining (n = 5). All data are presented as means ± SDs. ***p < 0.001 vs. control, as determined by Student’s *t*-test.

**Fig. S7 ME7 infection causes apoptotic cell death in PrP^C^-positive** **HSPCs.**

**A** Numbers of LSK cells and HSCs in the BM of old-aged control and ME7-infected mice were measured in relation to PrP^C^ expression (n = 6). **B** and **C** Levels of mitochondrial ROS and annexin V in PrP^C^-negative and -positive LSK cells and HSCs were assessed in old-aged control and ME7-infected mice (n = 6). **D** For competitive transplantation experiments, LSK cells from control or ME7-infected old-aged mice (CD45.2) were co-transplanted with equal numbers (5 × 10^3^) of LSK cells from competitor mice (CD45.1) and accessory cells (1 × 10^6^) from the PB of non-conditioned recipient mice (CD45.1/2) into conditioned recipient mice (CD45.1/2, 900 rads, n = 7); serial transplantation of BM cells (1 × 10^6^) of CD45.1/2 mice was performed after primary transplantation. The CD45.1/CD45.2 ratio in PB collected from recipient mice at 4 months post-transplantation was evaluated by flow cytometry. All data are presented as means ± SDs. *p < 0.05, **p < 0.01, and ***p < 0.001 vs. control, as determined by Student’s *t*-test.

**Fig. S8 ME7-infected old-aged mice exhibit no change in an apoptotic cell death-related factor.**

The percentage of annexin V-positive cells measured in PrP^C^-negative and -positive BM HSCs from control and ME7-infected old-aged mice (n = 4).

**Fig. S9 Expression of PrP^C^ protein in hematopoietic cells from CD45.1 competitor mice.**

PrP^C^ expression in phenotypically characterized hematopoietic cells, as mentioned in Fig. 1A, in the BM of CD45.1 competitor mice was examined using a biotinylated PrP^C^ antibody.

**Fig. S10 ME7 infection does not modulate the number of BM MSCs or their expression of apoptotic cell death-related factors.**

**A** The percentage of PrP^C^-positive BM MSCs (phenotypically defined as Lin^-^Sca-1^+^CD29^+^CD105^+^ cells) was assessed by multi-color flow cytometry. **B** The numbers of MSCs in the BM of control and ME7-infected mice were measured at the indicated times after infection (n = 7). **C** The percentages of annexin V-positive cells among PrP^C^-negative and PrP^C^-positive BM MSCs were measured in both mouse groups (n = 4); a representative result is shown.

**Fig. S11 ME7 infection does not influence osteogenesis.**

**A** and **B** Osteogenic differentiation (**A**) and mineralization (**B**) capacities of BM cells isolated from control and ME7-infected mice at multiple time points were examined for potent osteogenic signaling molecules including osteopontin (OPN), Runx2, and osterix (OSX) via western blotting, while mineralization activity was determined by alizarin red staining; representative results from three independent experiments are shown. **C** Femoral bone structures of ME7-infected old-aged mice were analyzed by micro-computed tomography imaging (n = 4); a representative result is shown. BMD, bone mineral density; BV/TV, bone volume/tissue volume; Tb.Th, trabecular thickness; Tb.N, trabecular number; Tb.Sp, trabecular separation.

**Fig. S12 Generation of *Prnp*** **knockout mice.**

**A** Genotyping of *Prnp* knockout mice. Upper panel shows PCR results for the wild-type allele of the *Prnp* gene (388 bp). Lower panel shows PCR results for the mutant allele of the *Prnp* gene (341 bp). +/+: homozygote with wild-type alleles of the *Prnp* gene; +/-: heterozygote with wild-type and mutant alleles of the *Prnp* gene; -/- homozygote with mutant alleles of the *Prnp* gene. **B** The expression of PrP^C^ was measured in BM-conserved hematopoietic cells from WT and KO mice.

**Fig. S13 Generation of *Prnp*** **knockout and wild-type mouse model of prion disease through i.p. injection of the ME7 scrapie strain.**

**A** Measurement of the body weights of ME7-infected WT and KO mice at 5 and 7 months post-injection. **B** Western blotting was performed to evaluate PrP^Sc^ bands in homogenates of brain tissue and homogenates of BM cells from control and ME7-infected *Prnp* knockout and wild-type mice at 5 months post-injection; a representative result is shown. Upper gel shows western blot bands of PrP^Sc^ in homogenates of brain tissue from *Prnp* knockout and wild-type mice. Lower gel shows western blot bands of PrP^Sc^ in homogenates of BM cells from *Prnp* knockout and wild-type mice. **C** Western blotting was performed to evaluate PrP^Sc^ bands in homogenates of brain tissue and homogenates of BM cells from control and ME7-infected *Prnp* knockout and wild-type mice at 7 months post-injection; a representative result is shown. Upper gel shows western blot bands of PrP^Sc^ in homogenates of brain tissue from *Prnp* knockout and wild-type mice. Lower gel shows western blot bands of PrP^Sc^ in homogenates of BM cells from *Prnp* knockout and wild-type mice. HSP90 was used as a loading control. All data are presented as means ± SDs. ***p < 0.001 vs. WT or KO without ME7, as determined by Student’s *t*-test.

**Fig. S14 ME7-mediated modulation of HSCs and the BM microenvironment is triggered by PrP^C^-based signaling.**

**A-G** Levels of mitochondrial ROS (**A**, n = 5), annexin V (**B**, n = 5), caspase 3 (**C**, n = 5), SA-β-gal activity (**D**, n = 5), p16 expression (**E**, n = 5), p-JNK (**F**, n = 5), and p-p38 (**G,** n = 5) were analyzed in BM HSCs from WT and KO mice with and without ME7 infection at the indicated times. (**H**) Clonogenic formations of CFU-EM, BFU-E, CFU-GEMM, and pre-B cells were counted after incubation of BM cells from WT and KO middle-aged mice with and without ME7. Representative data from three independent experiments are shown. (**I**), SA-β-gal activities were measured in BM MSCs from WT and KO mice with and without ME7 at the indicated times (n = 5). **J-M** The adiponectin level (**J**, n = 5), PPARγ level (**K**, n = 5), osteoclast activity (**L**, n = 5), and RANKL level (**M**, n = 5) were measured in the BM of WT and KO mice with and without ME7 infection; a representative result is shown. Scale bars are 200 µm. All data are presented as means ± SDs. *p < 0.05, **p < 0.01, and ***p < 0.001 vs. WT or KO without ME7, as determined by Student’s *t*-test.

**Fig. S15 Hematopoiesis in WT and KO mice with and without ME7.**

A complete blood count in the PB of WT and KO mice with and without ME7 infection were assessed using an automated complete cell counter (n = 5). All data are presented as means ± SDs. *p < 0.05, **p < 0.01, and ***p < 0.001 vs. WT or KO without ME7, as determined by Student’s *t*-test.

**Video S1 Video frames of control and ME7-infected mice.**

**A** Video frame of control mice at 5 months post-injection. **B** Video frame of prion-infected mice at 5 months post-injection. **C** Video frame of control mice at 7 months post-injection. **D** Video frame of prion-infected mice at 7 months post-injection.

**Video S2 Video frames of ME7- and non-infected *Prnp* WT and KO mice.**

**A** Video frame of WT mice at 5 months post-injection. **B** Video frame of prion-infected WT mice at 5 months post-injection. **C** Video frame of KO mice at 5 months post-injection. **D** Video frame of prion-infected KO mice at 5 months post-injection. **E** Video frame of WT mice at 7 months post-injection. **F** Video frame of prion-infected WT mice at 7 months post-injection. **G** Video frame of KO mice at 7 months post-injection. **H** Video frame of prion-infected KO mice at 7 months post-injection.

**Table S1 Primer sequences used in this study**

| Gene | Forward sequence | Reverse sequence |
| --- | --- | --- |
| *p15* | CCCTGCCACCCTTACCAGA | CAGATACCTCGCAATGTCACG |
| *p16* | GTCGCAGGTTCTTGGTCACT | TCTGCACCGTAGTTGAGCAG |
| *p19* | GCCGGCAAATGATCATAGAG | CAGCAAGAGCTGGATCAGAA |
| *p21* | TGTCCGTCAGAACCCATC | AAAGTCGAAGTTCCATCGCC |
| *GAPDH* | GACGGCCGCATCTTCTTGT | CACACCGACCTTCACCATTTT |
| *PRNP-*wild type | GATCCATTTTGGCAACGACT | GAGAATGCGAAGGAACAAGC |
| *PRNP-*mutant | GCATCGCCTTCTATCGCC |  |
