# Supplementary figures and images for: Prion infection modulates hematopoietic stem/progenitor cell fate through cell-autonomous and non-autonomous mechanisms
Source: Leukemia. 2023 Jan 27;37(4):877–87. doi: 10.1038/s41375-023-01828-w (PMC10079512; doi:10.1038/s41375-023-01828-w)

# A

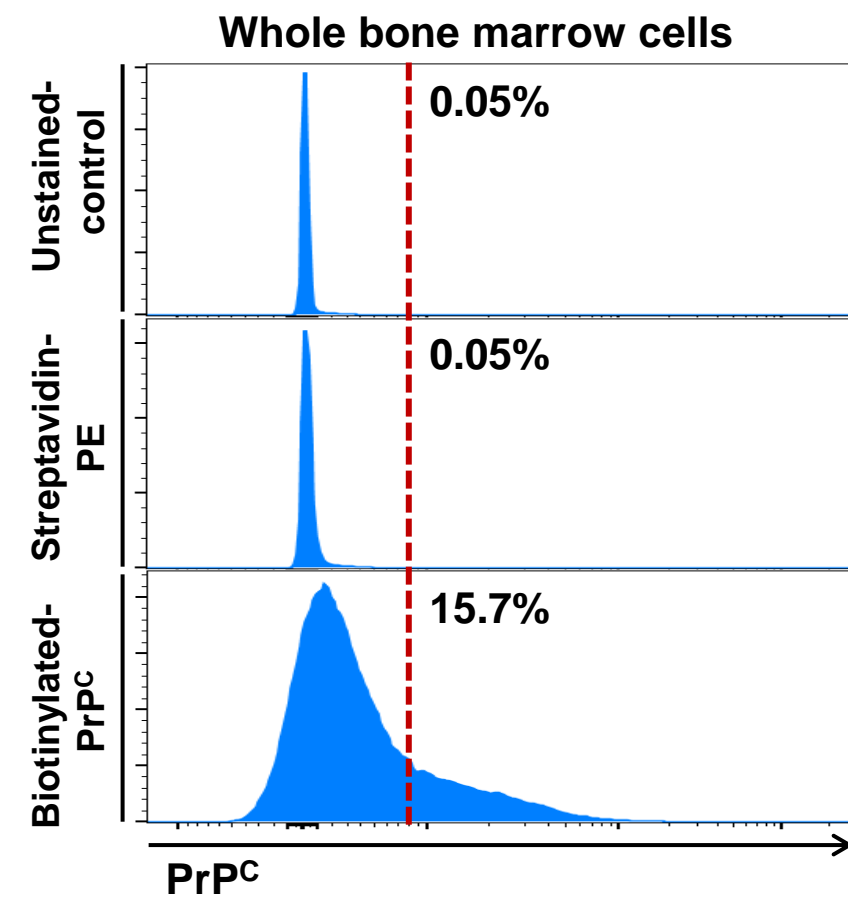

# B

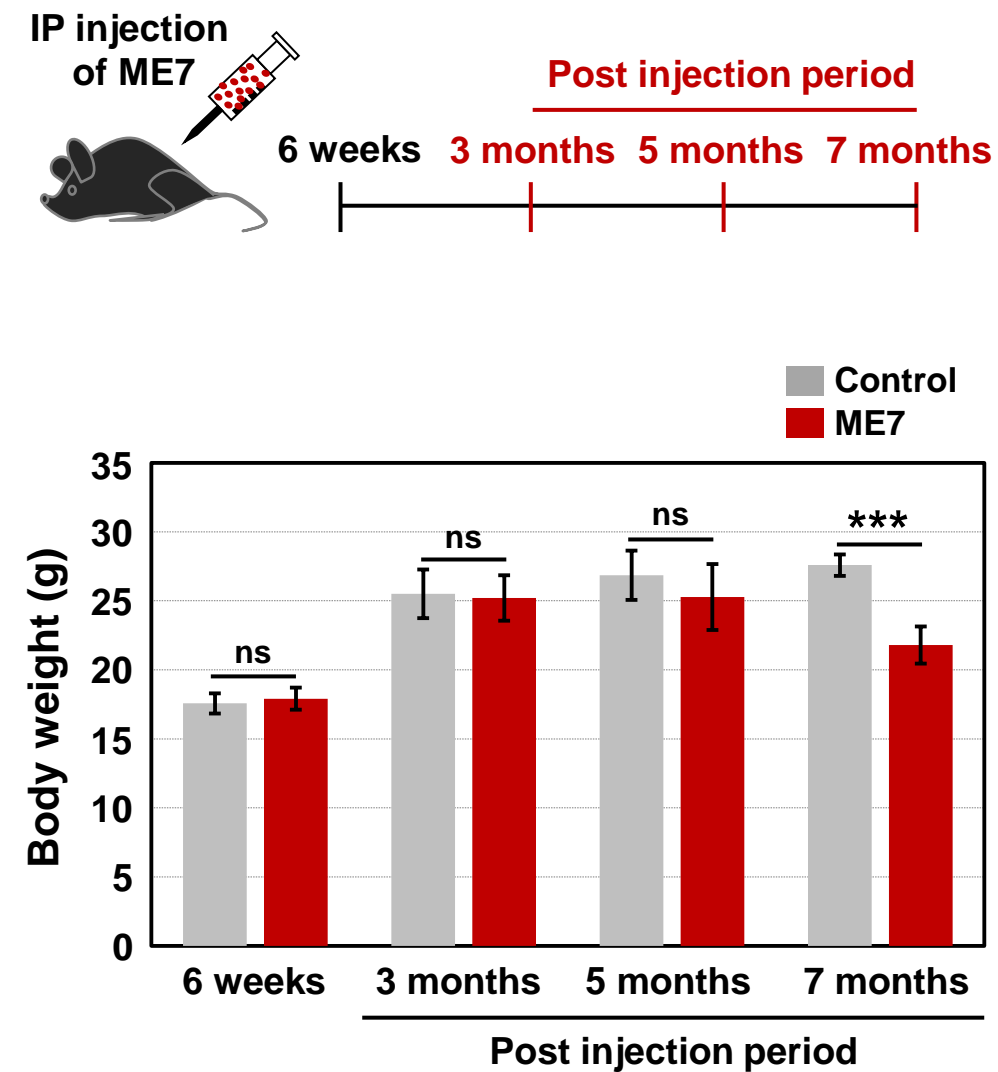

# C

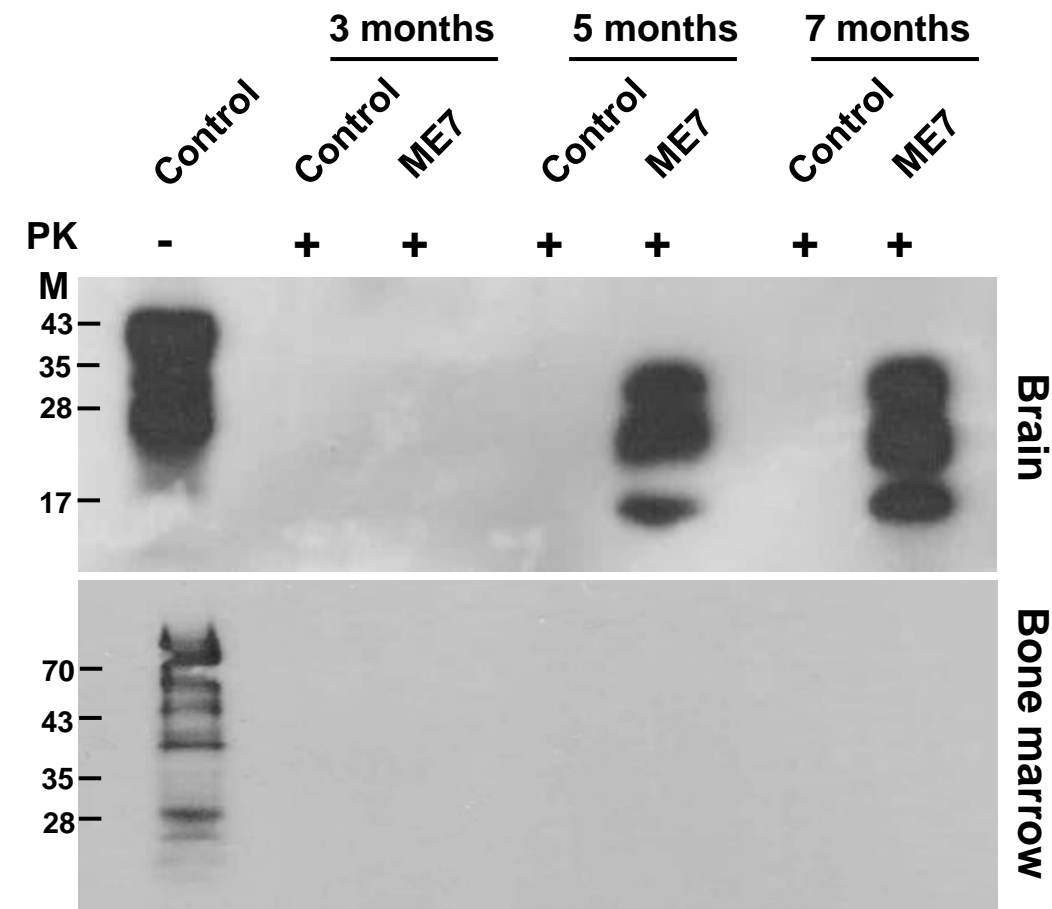

# D

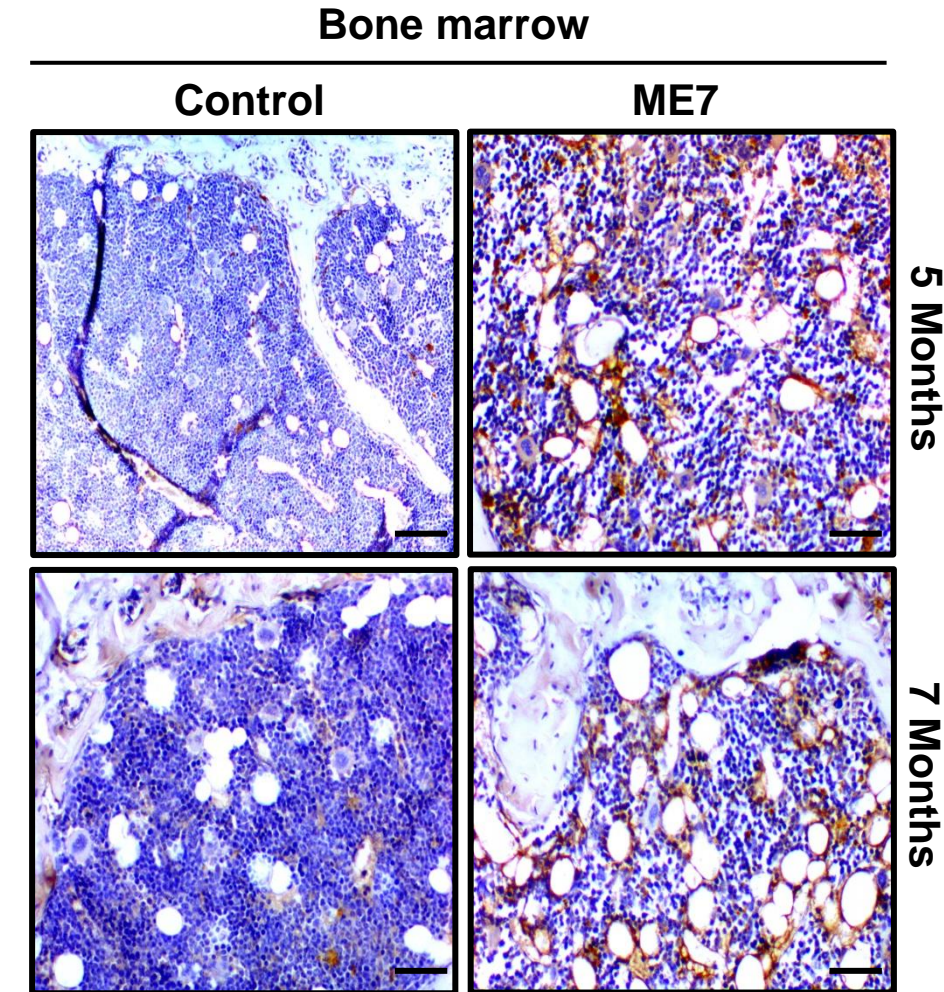

**A****1<sup>st</sup> passage (5 months)**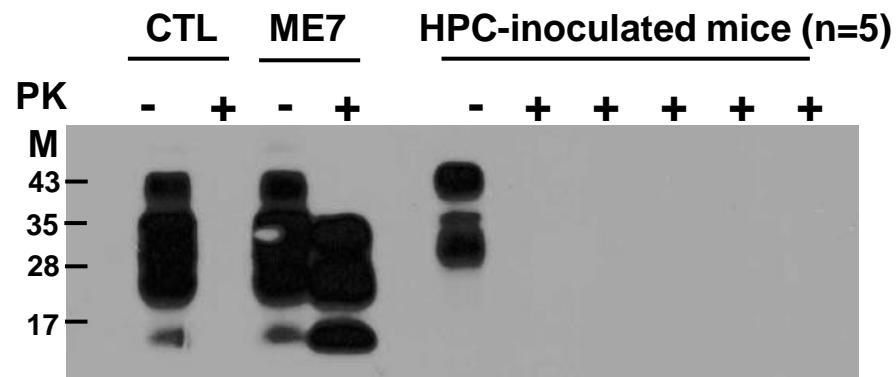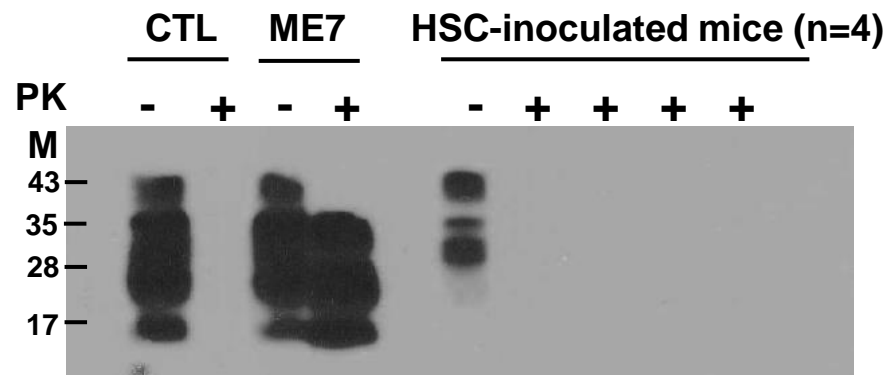**B****2<sup>nd</sup> passage (5 months)**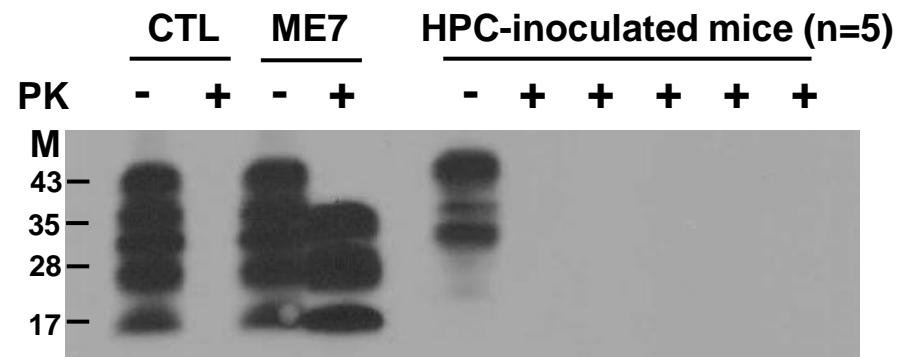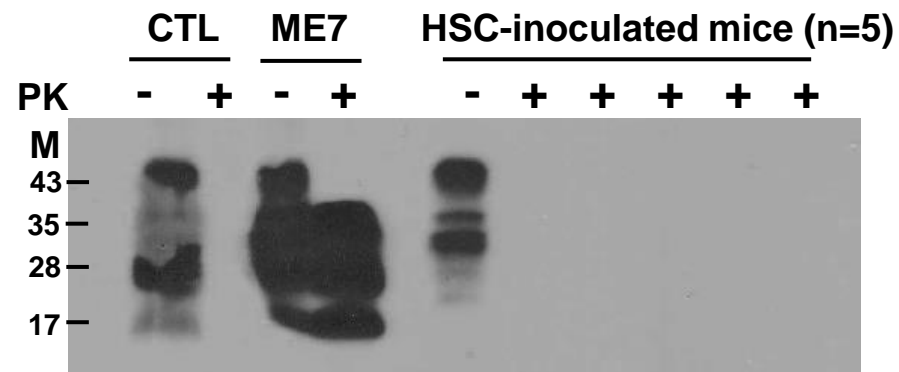

**A**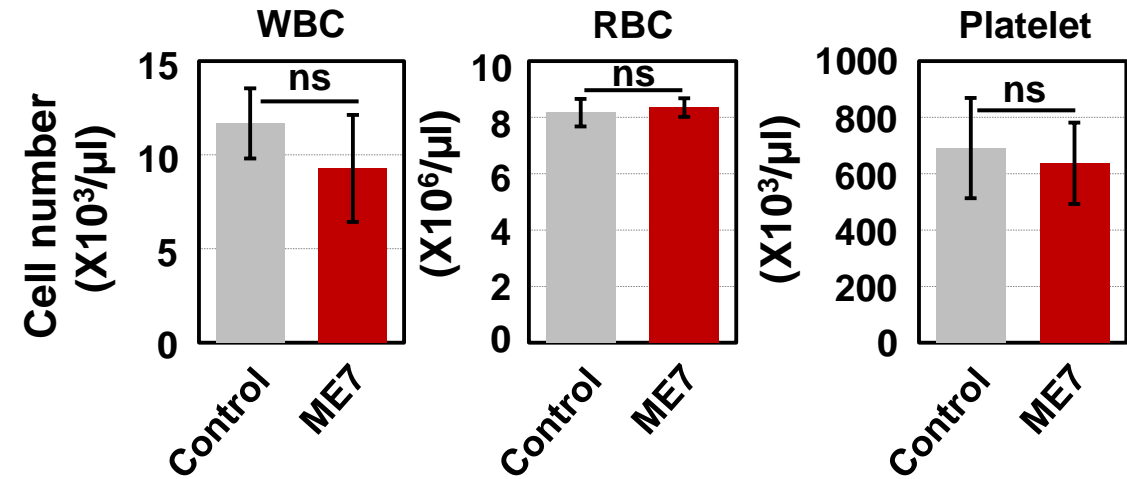**B**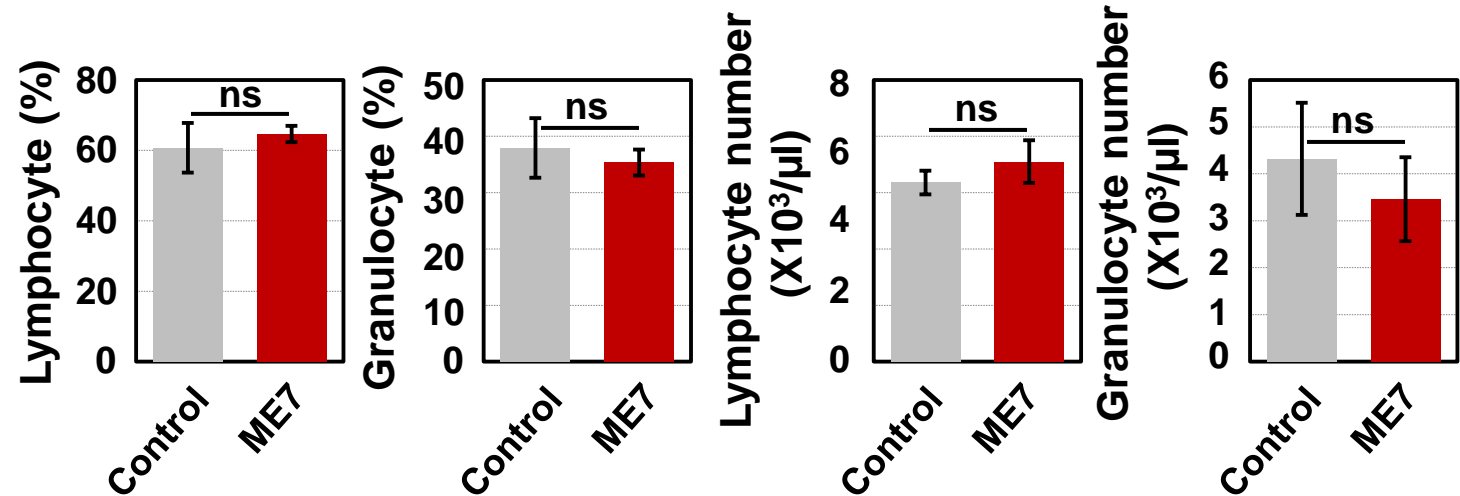**C**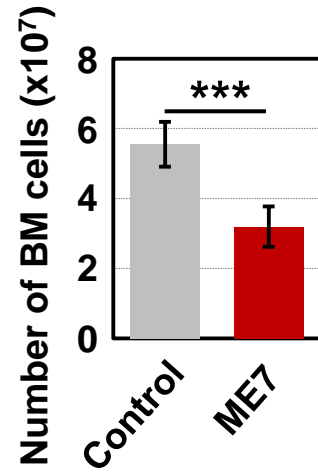

**A**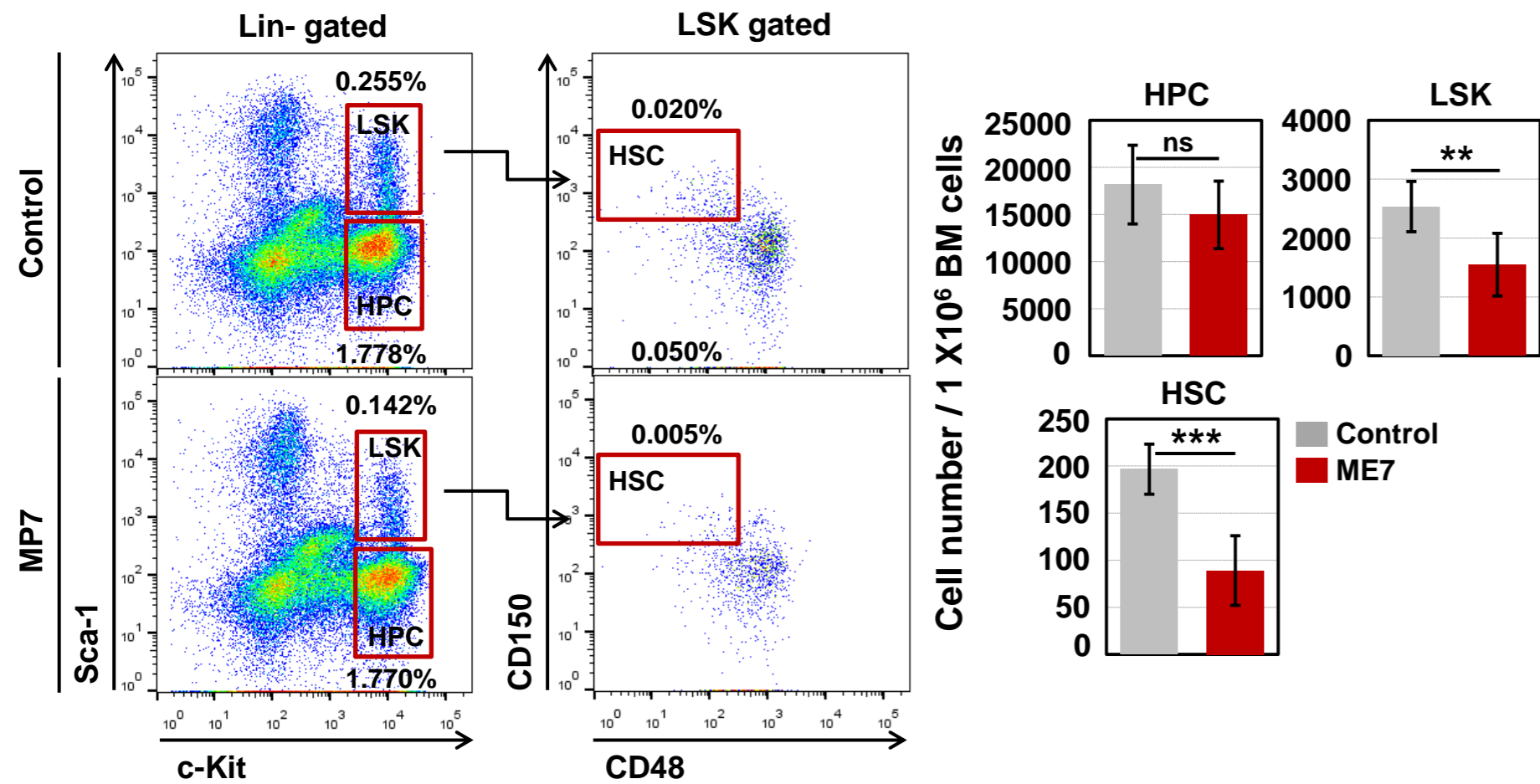**B**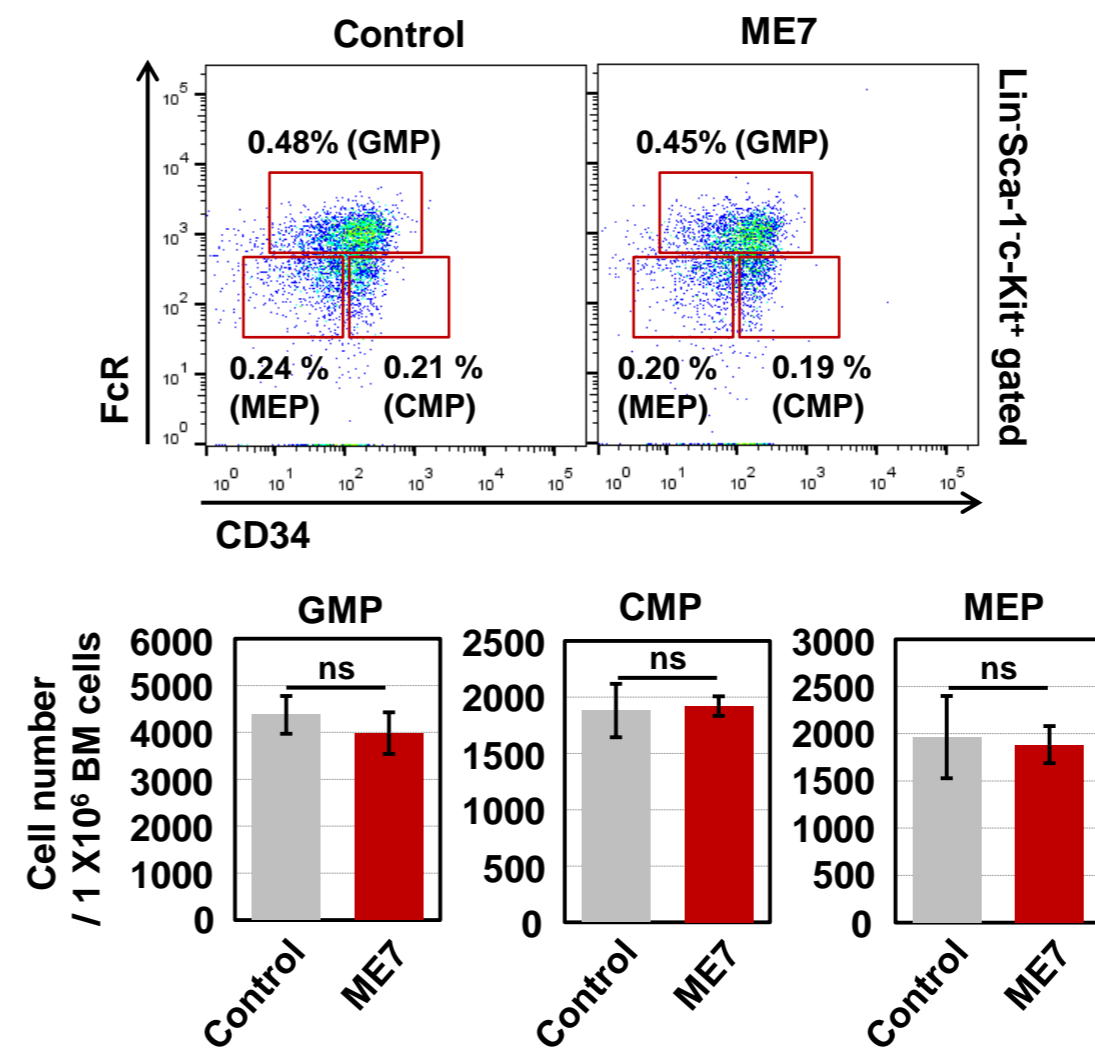**C**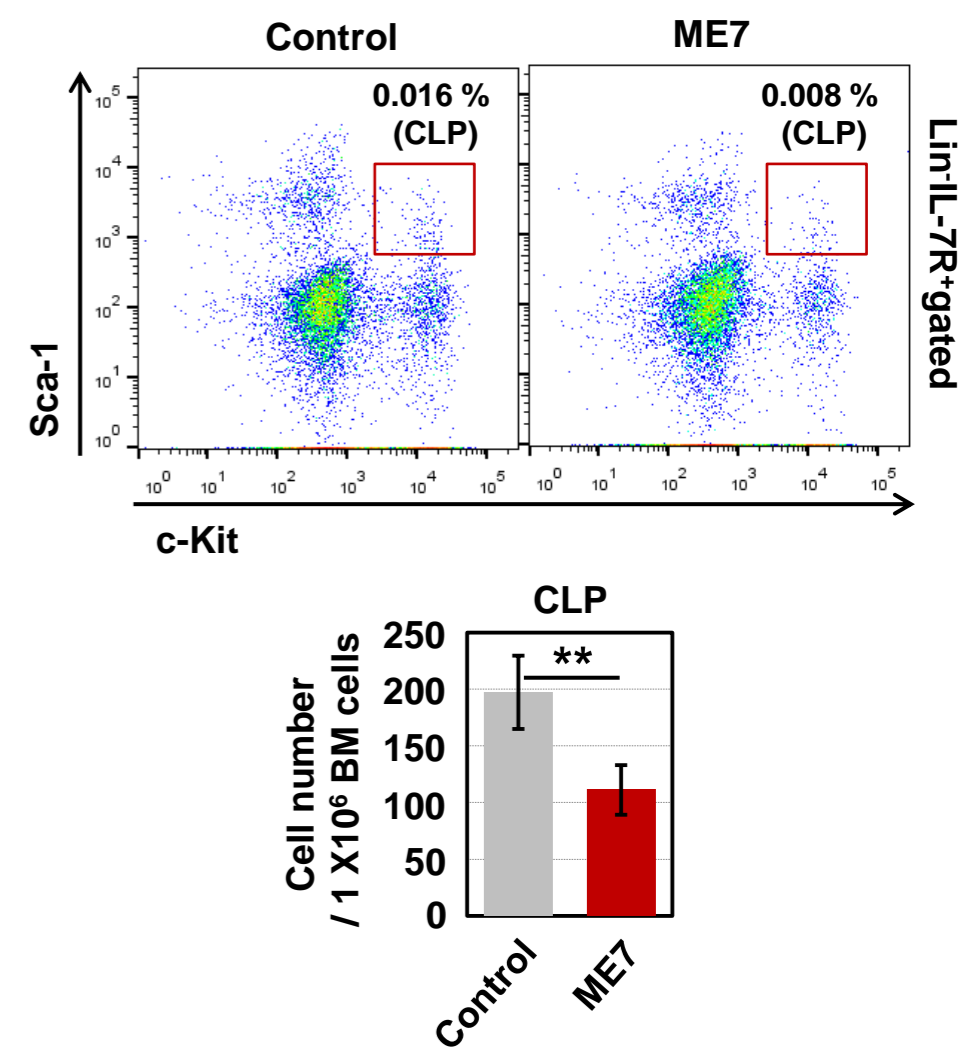

**A**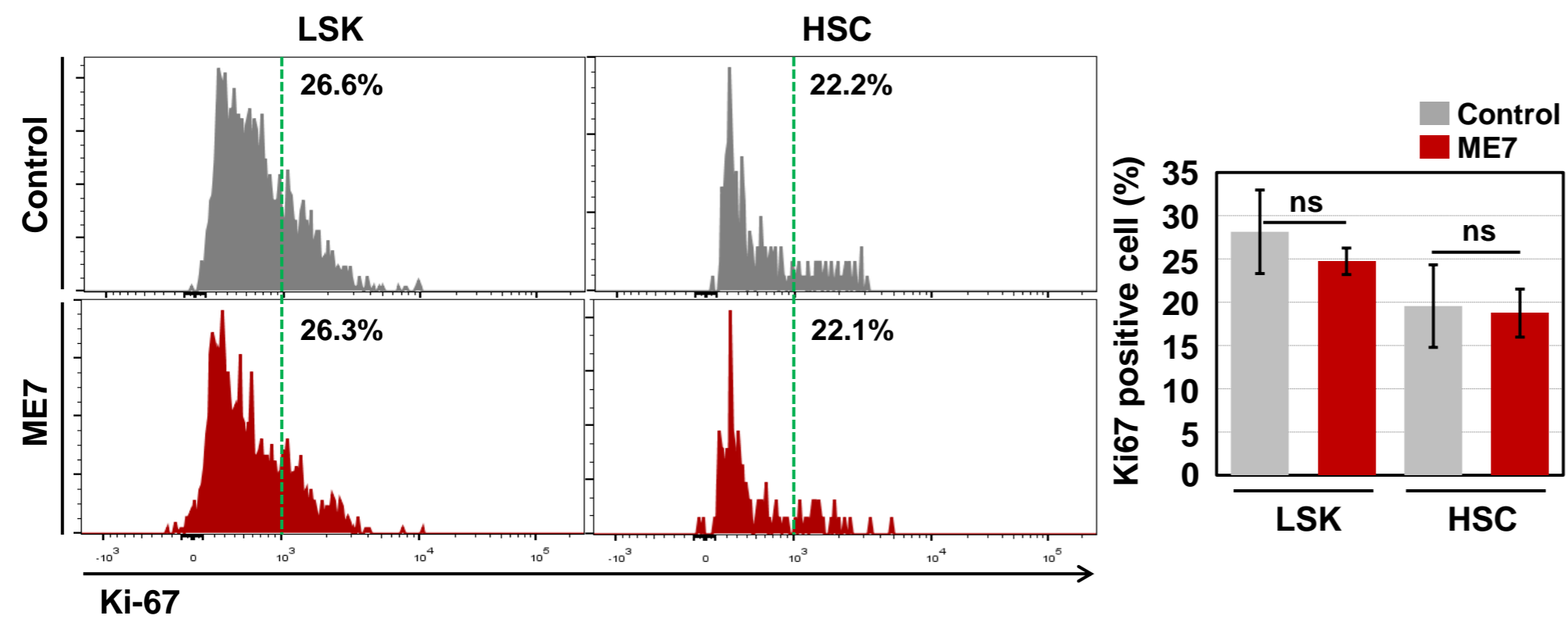**C**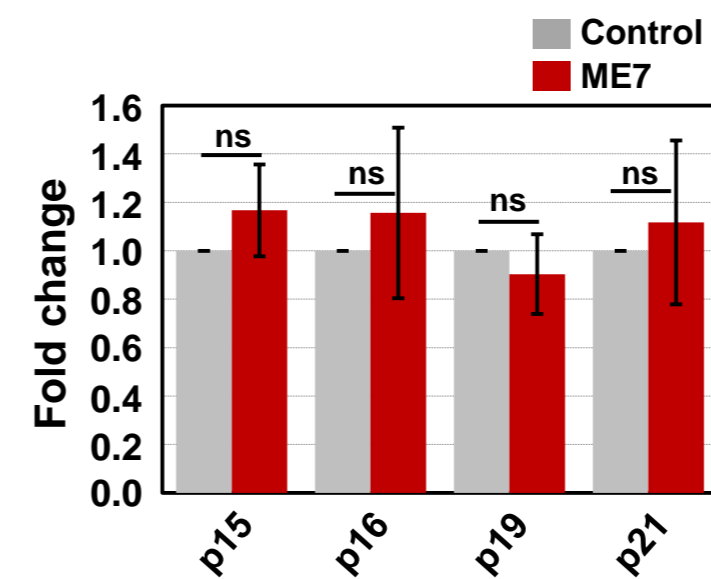**B**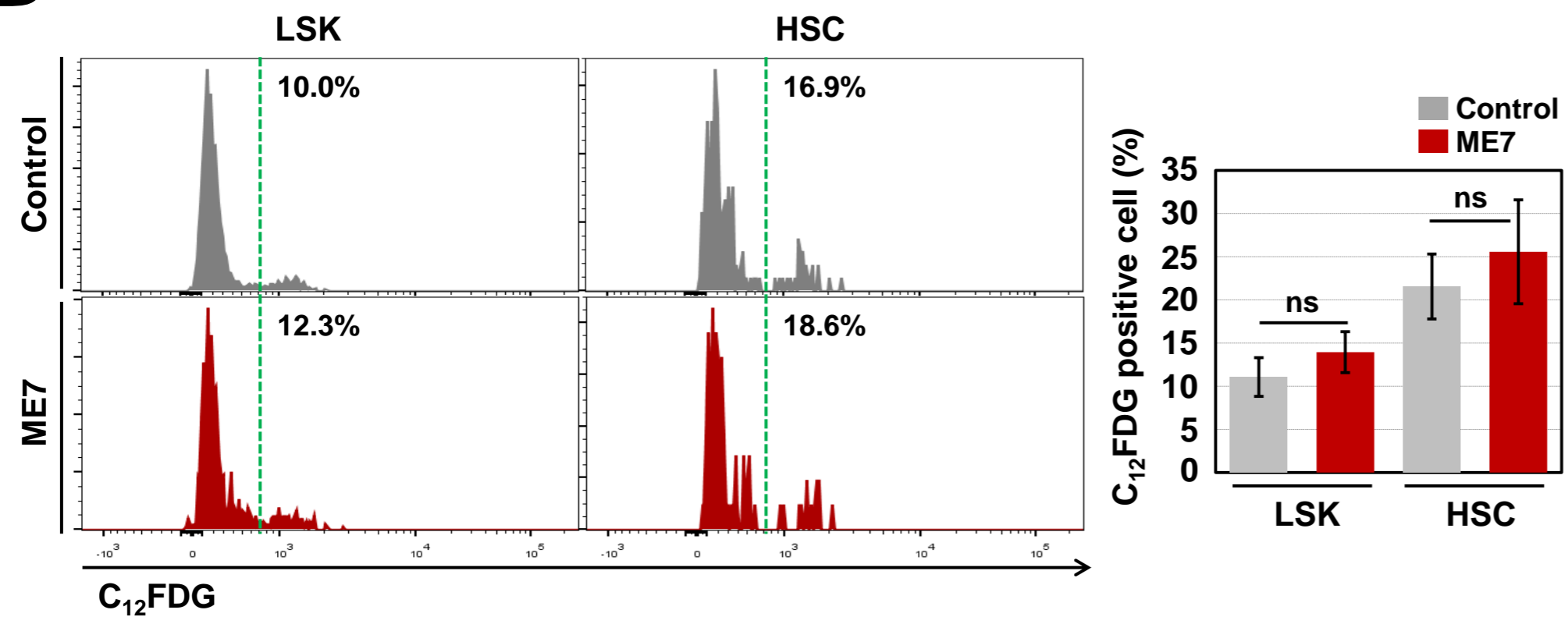**D**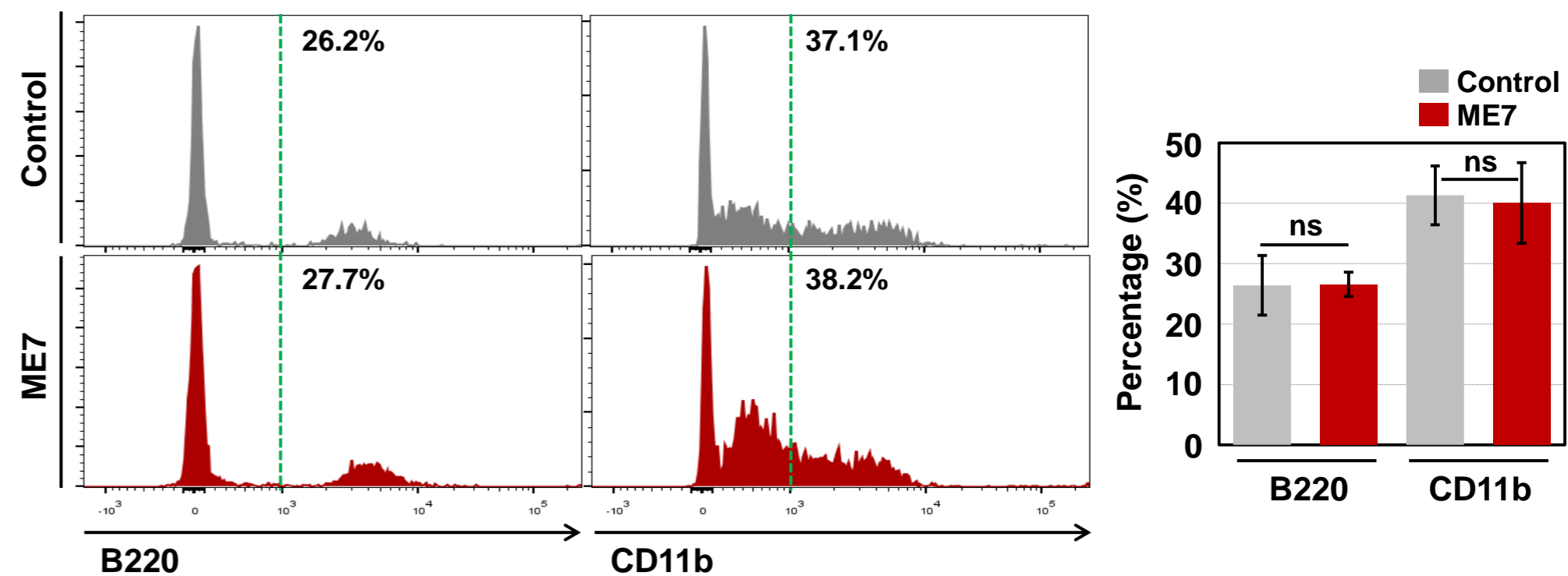

**A**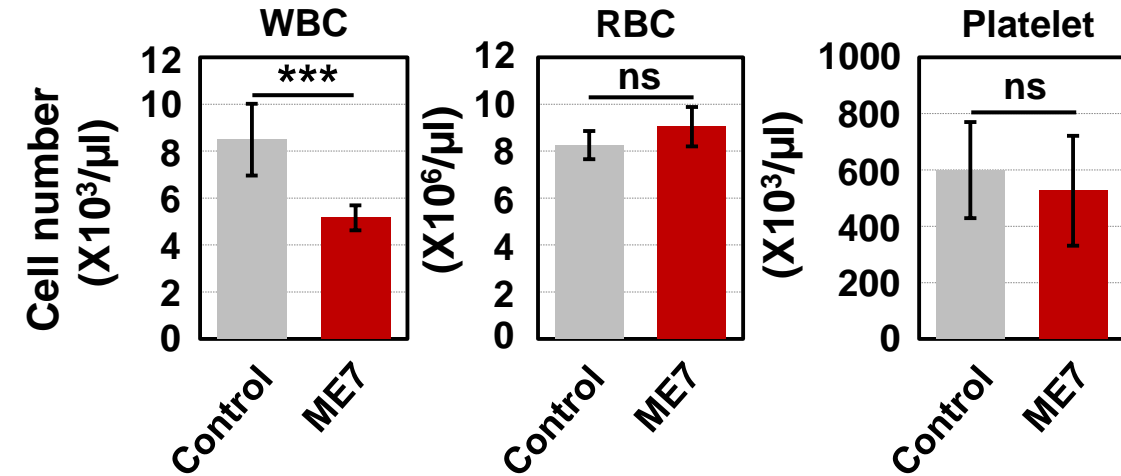**B**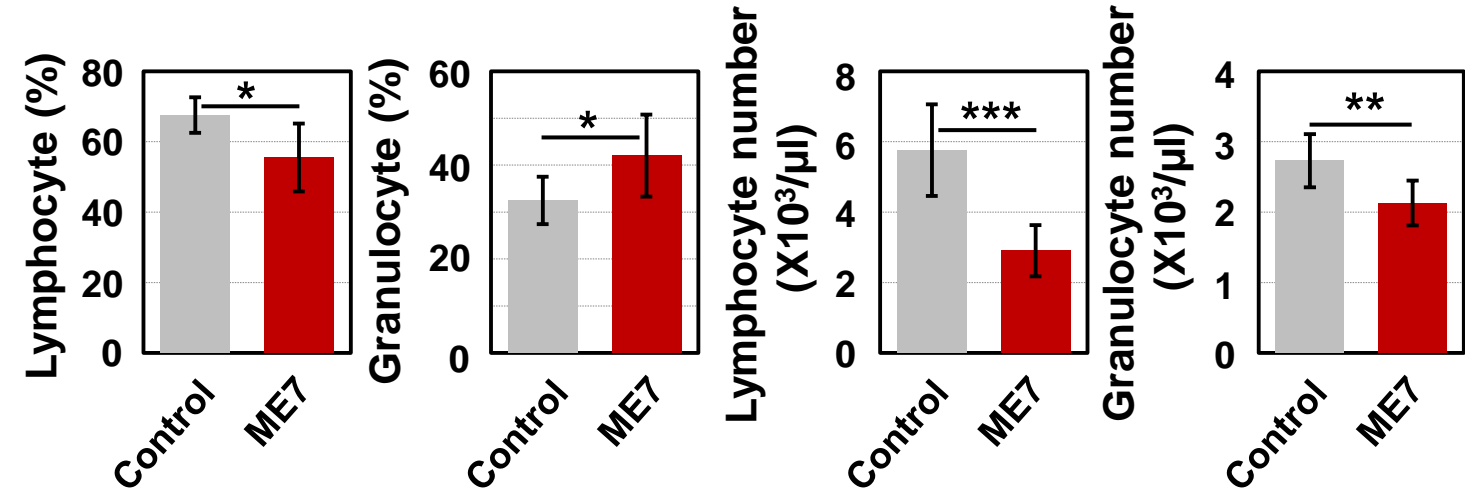**C**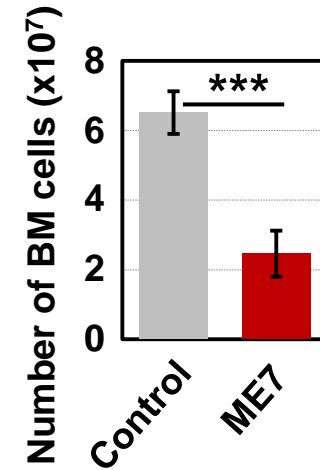

**A**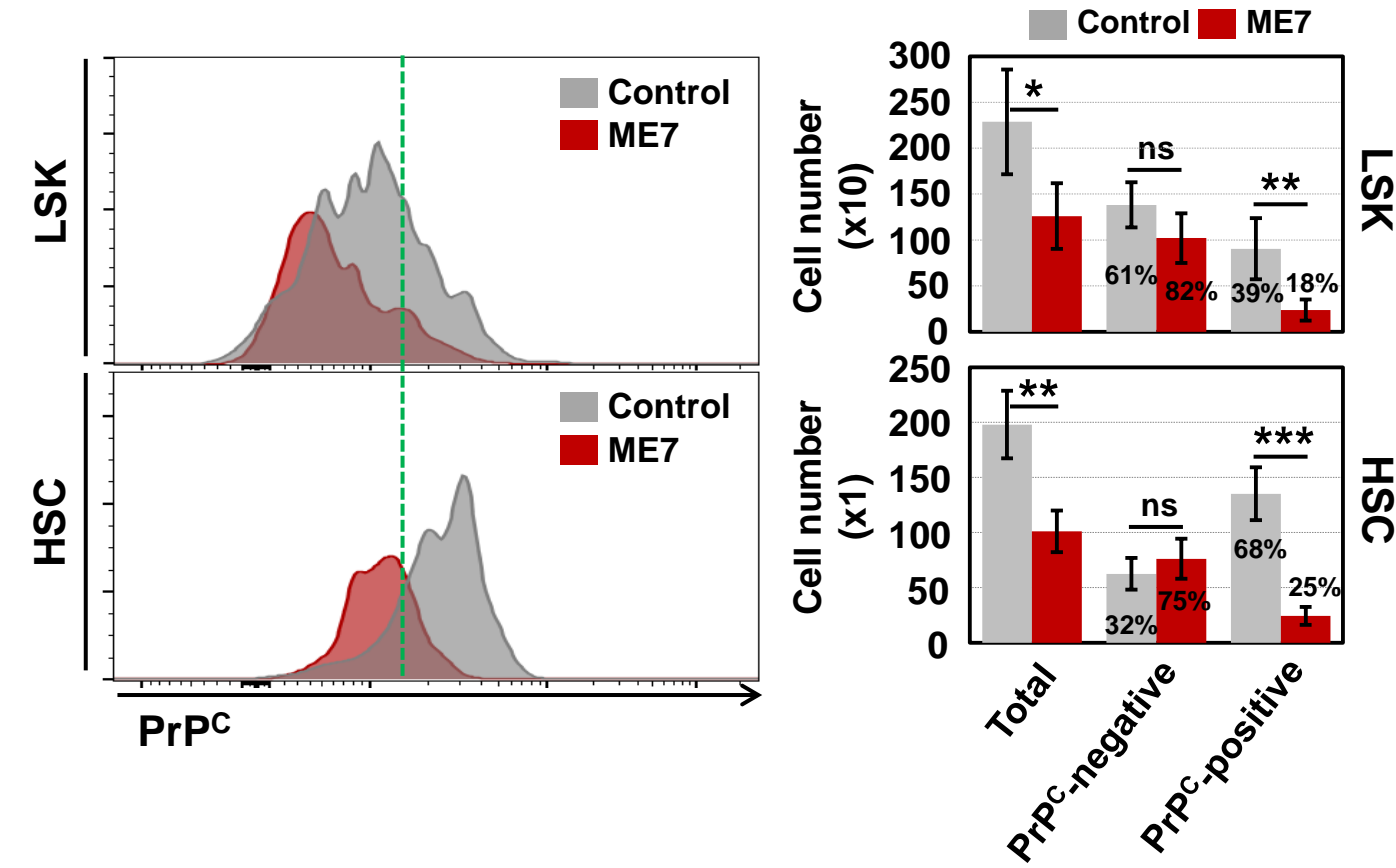**B**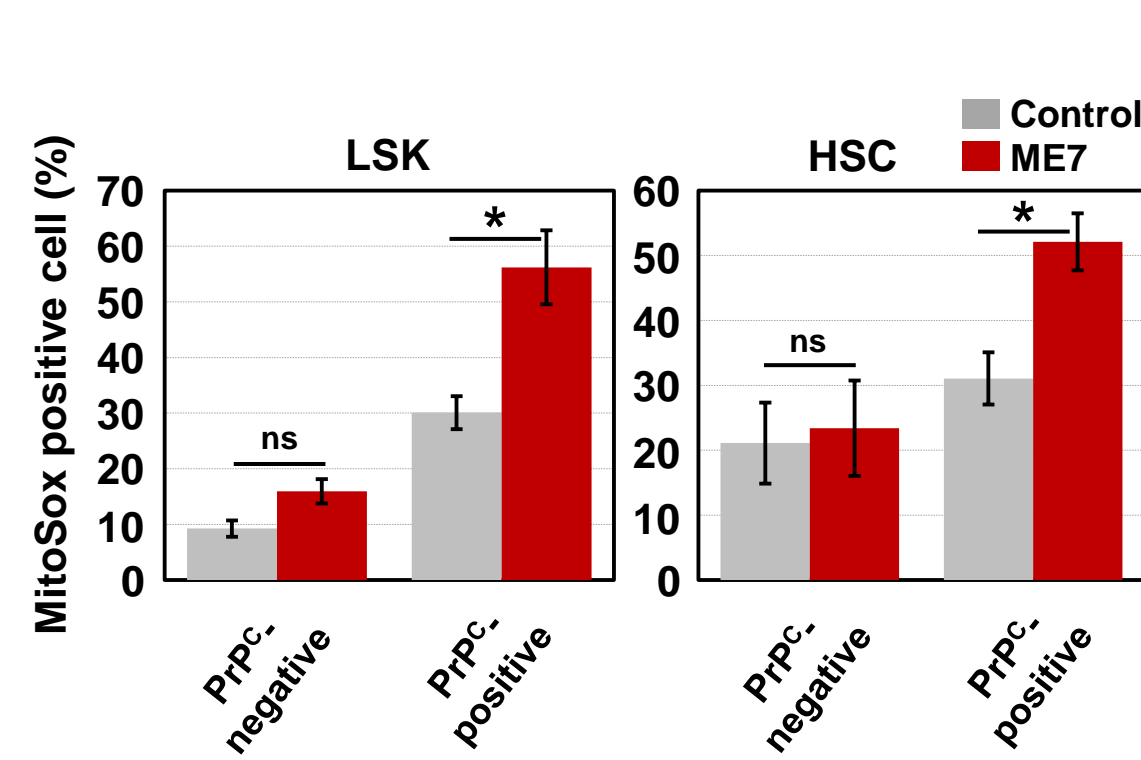**C**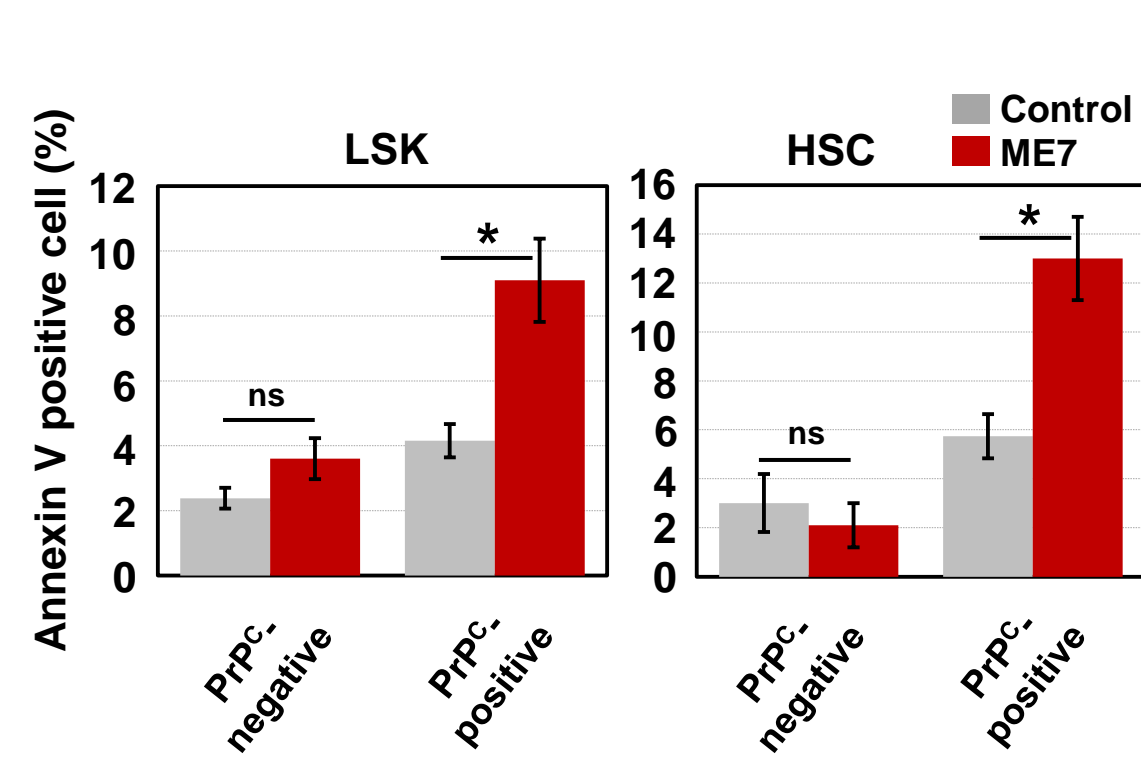**D**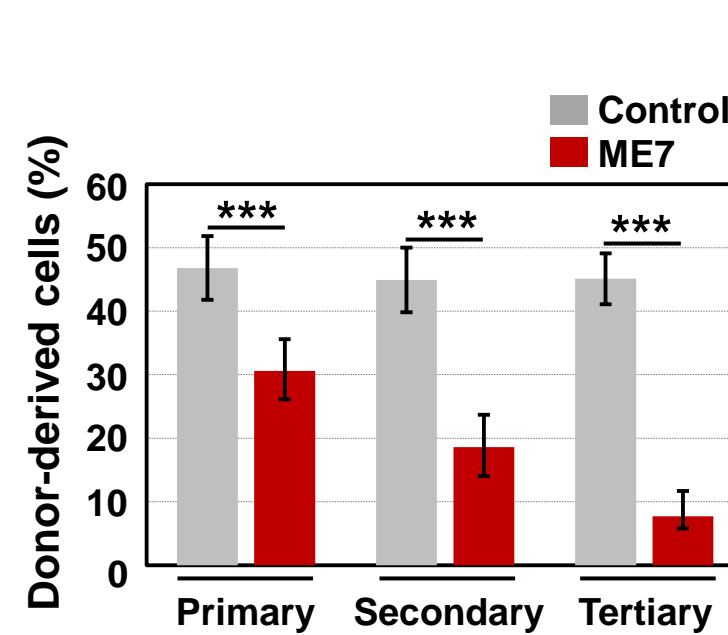

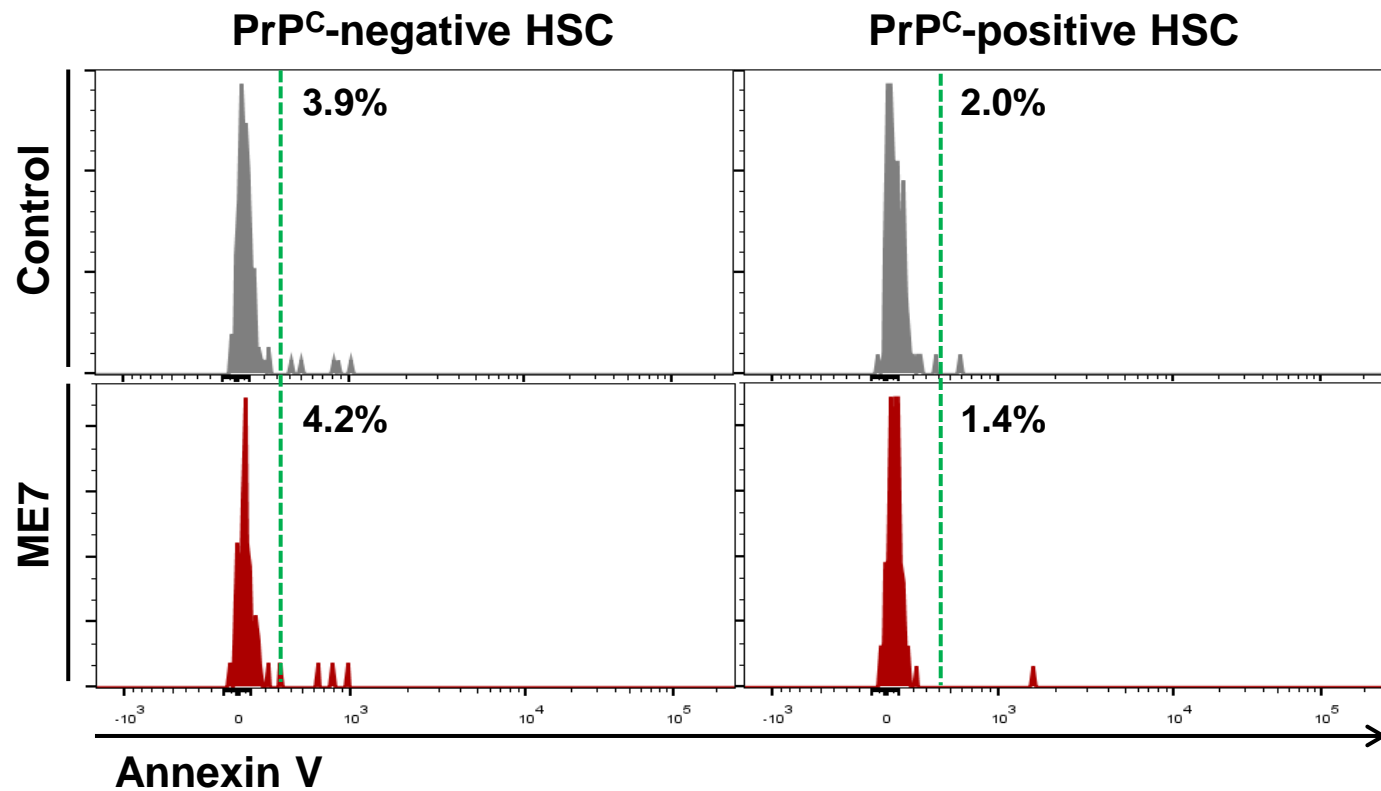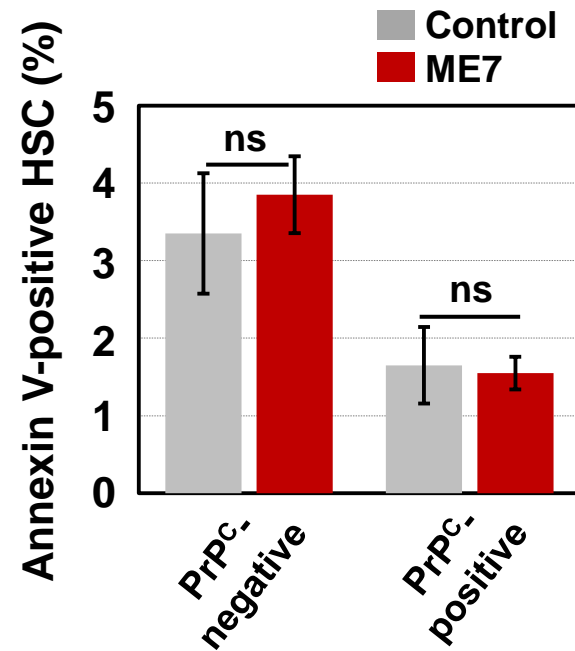

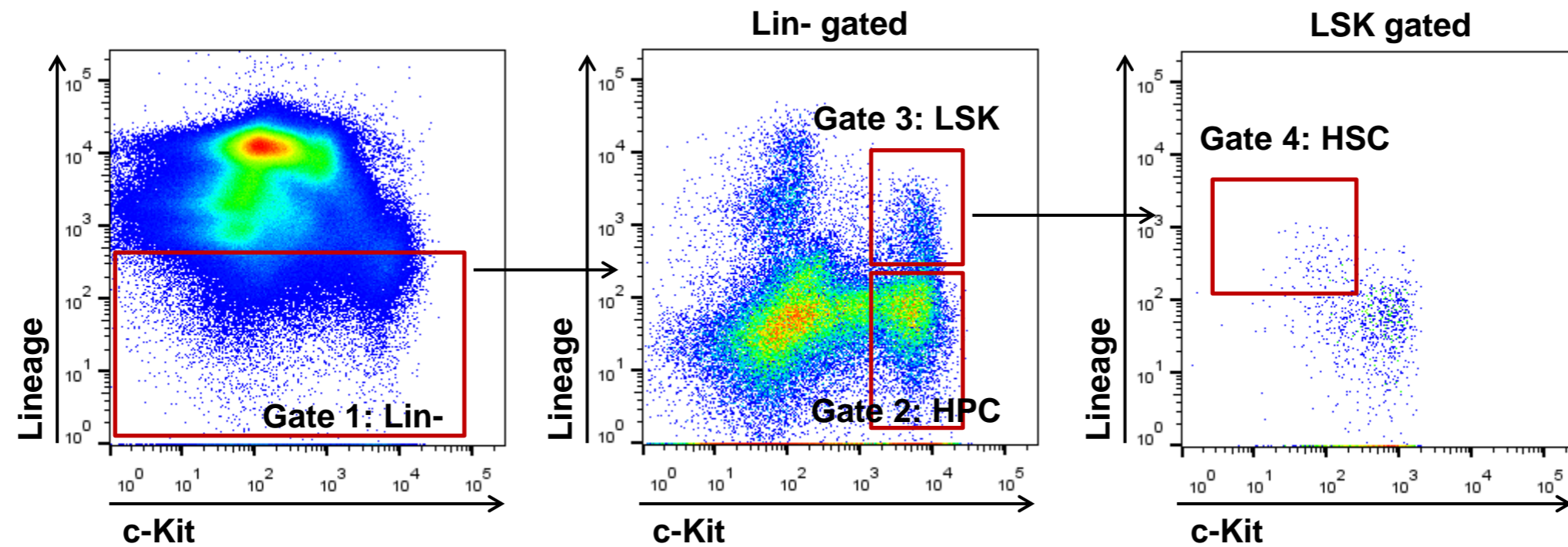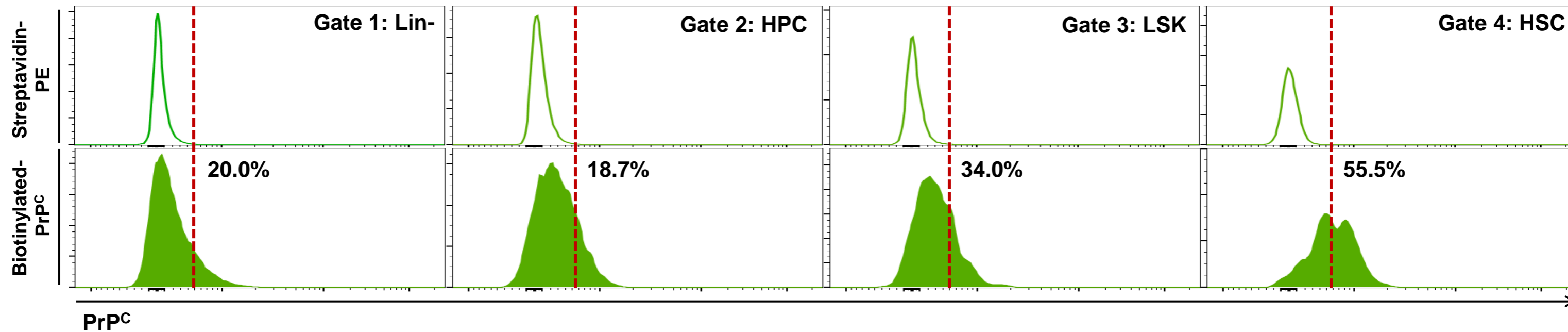

**A**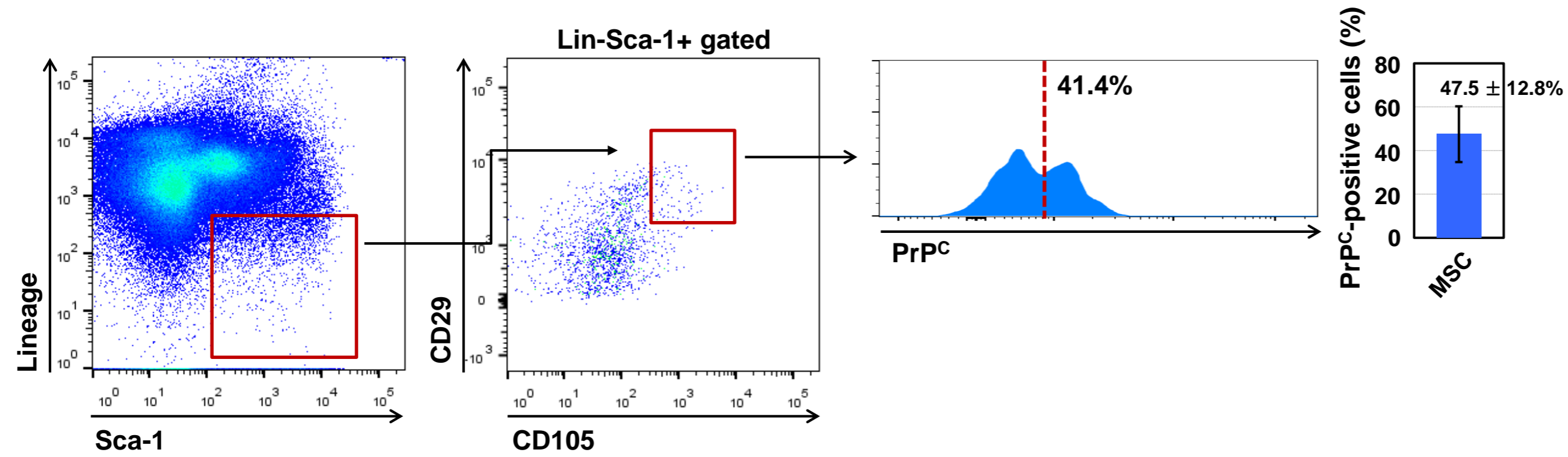**B**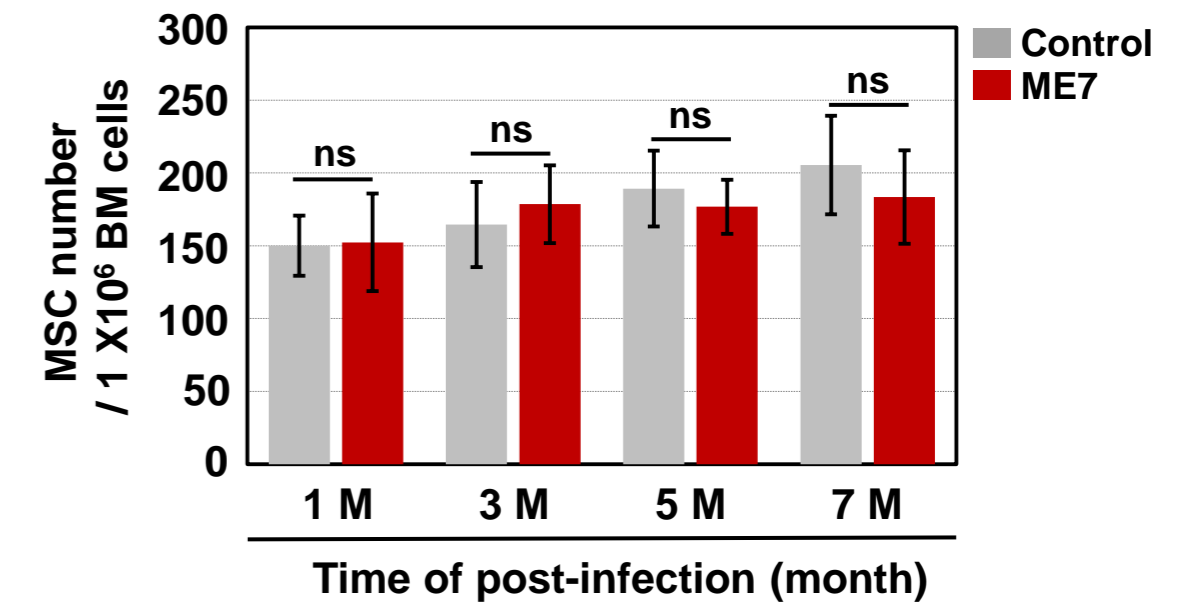**C**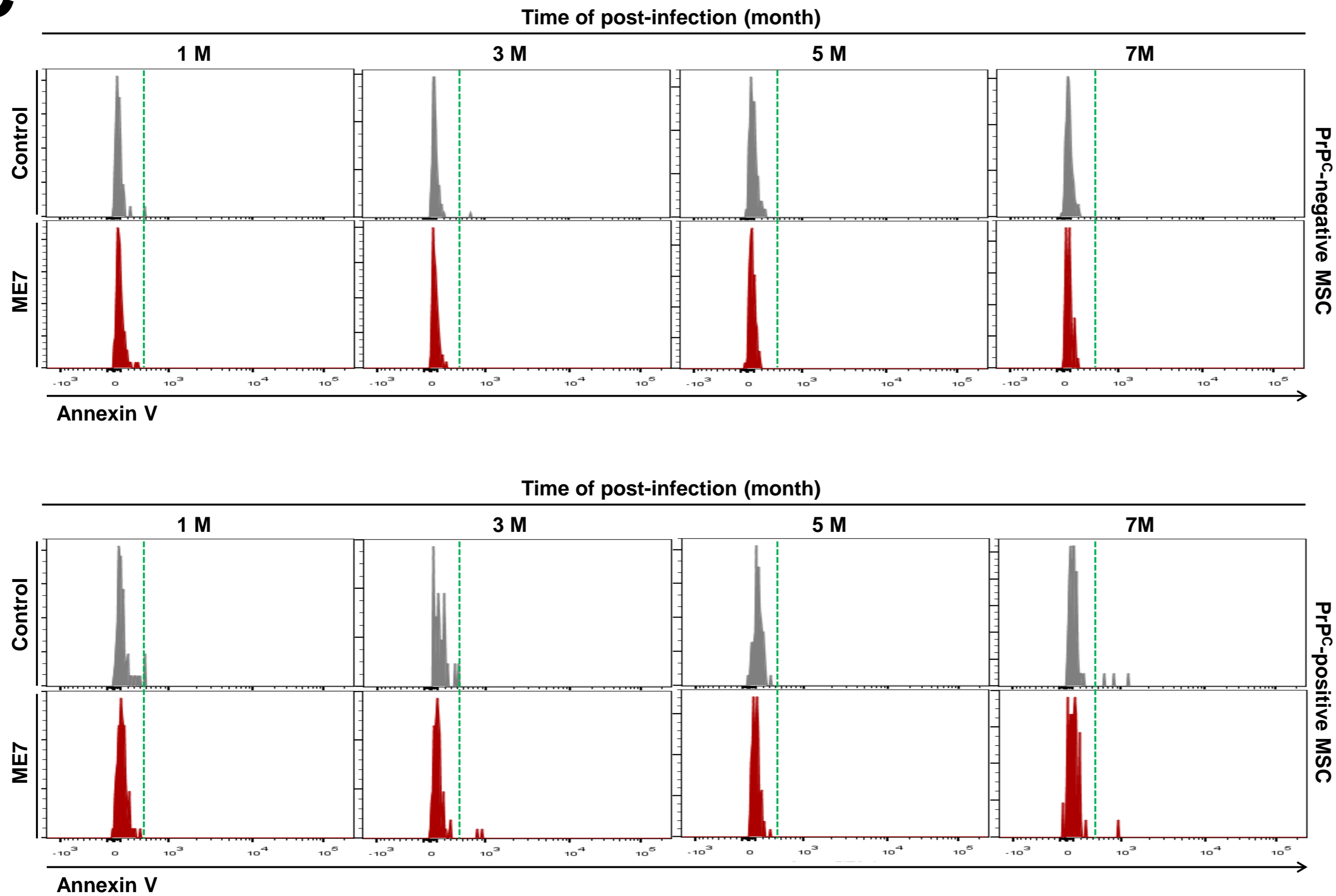

**A**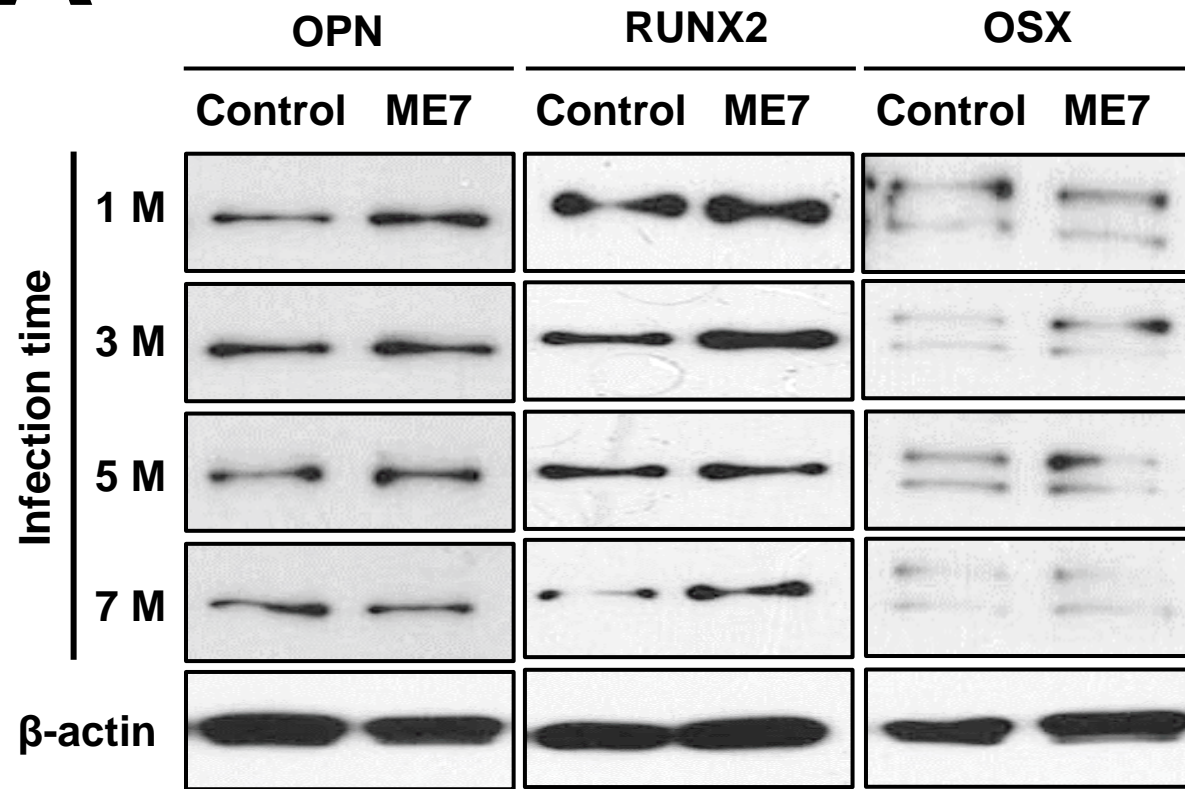**B**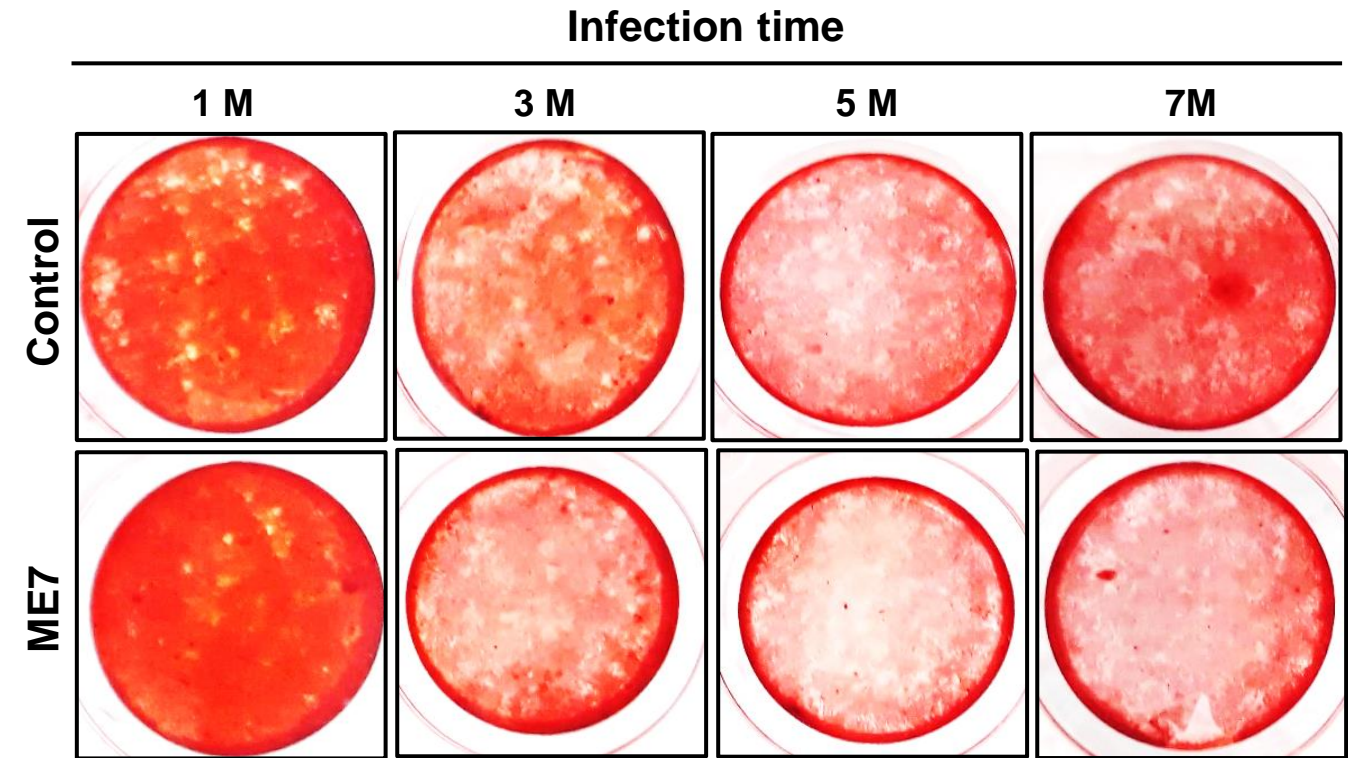**C**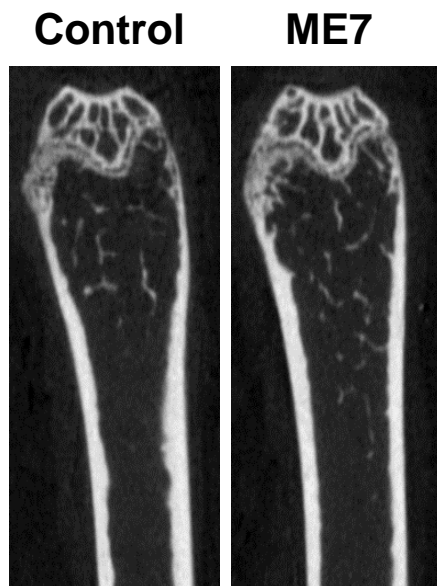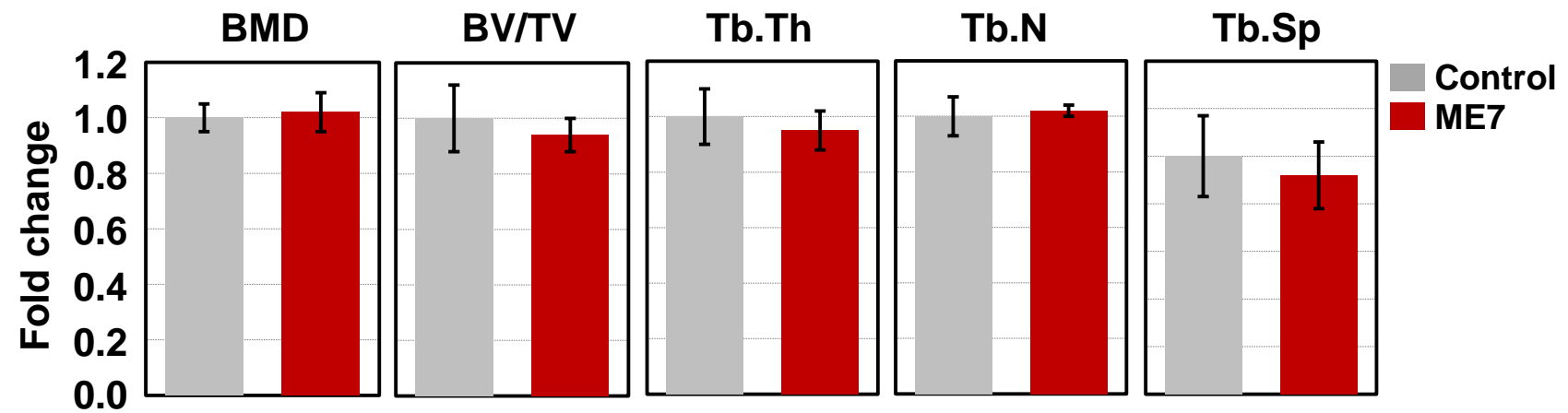

**A**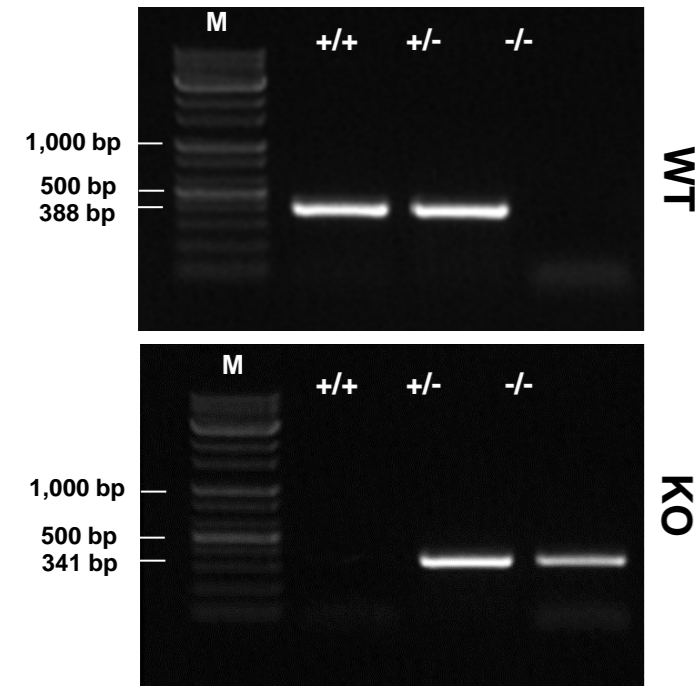**B**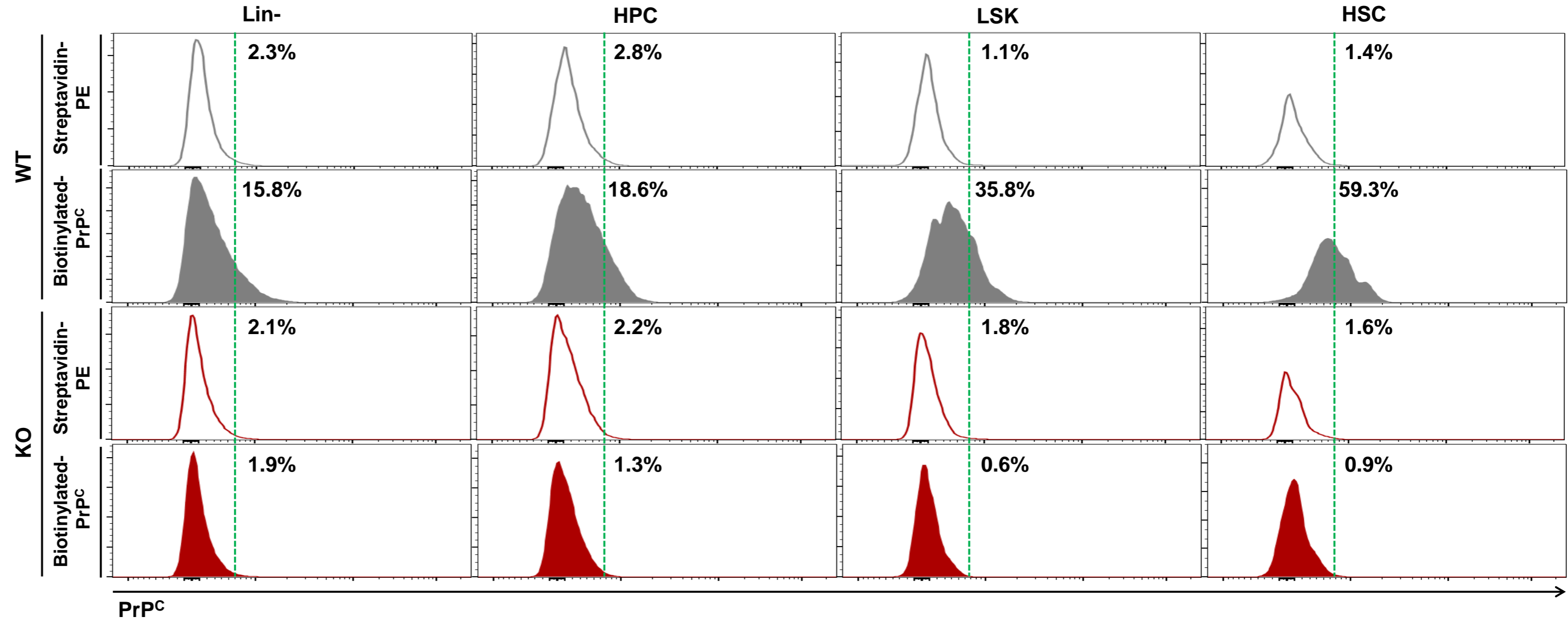

**A**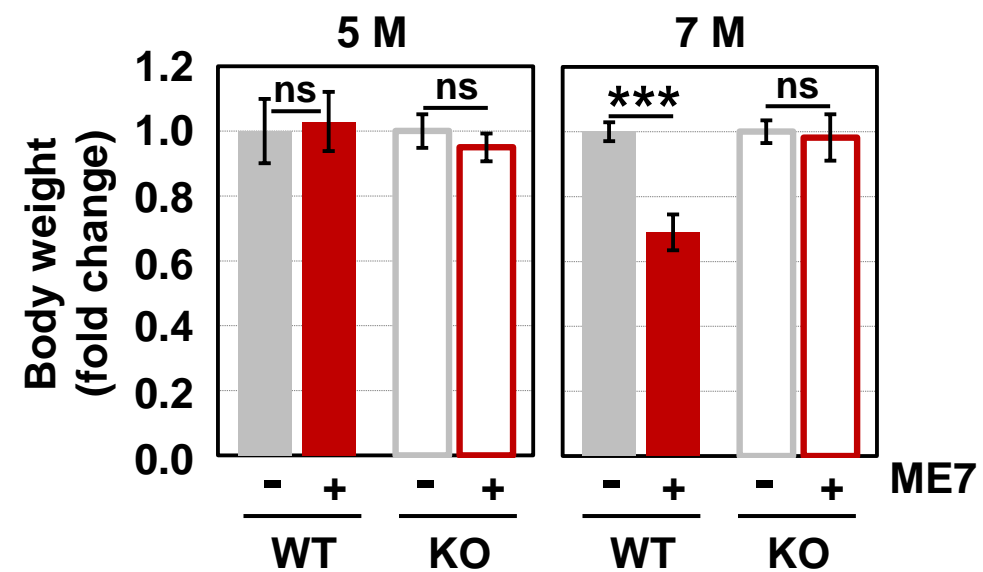**B**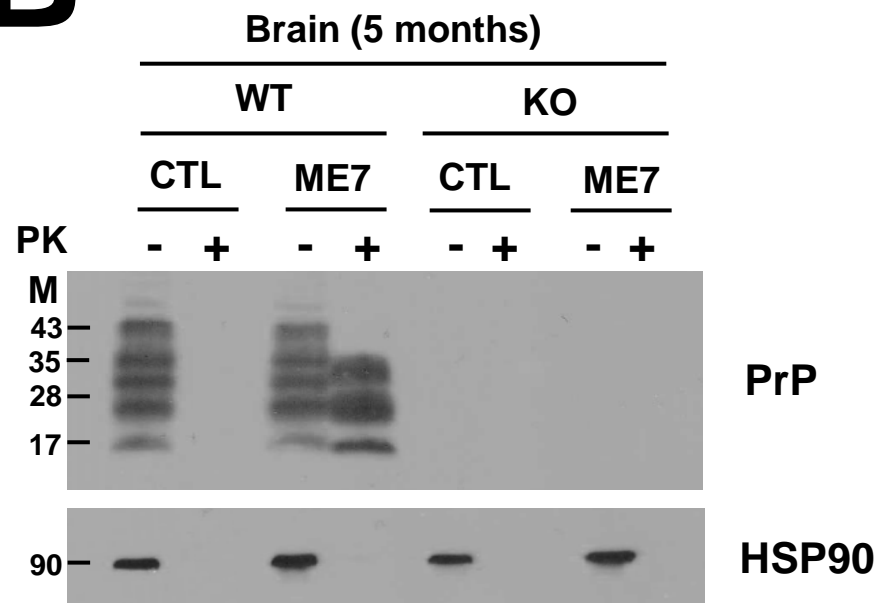**C**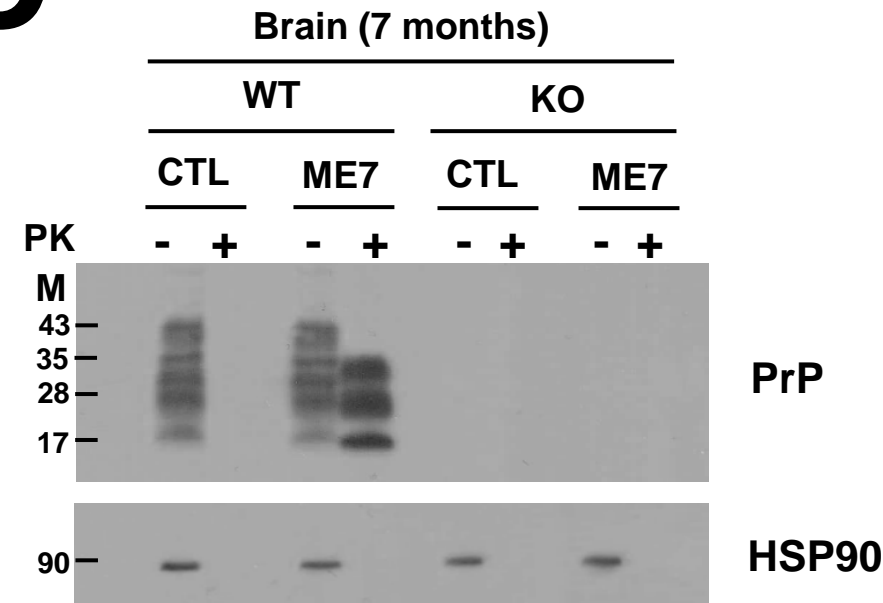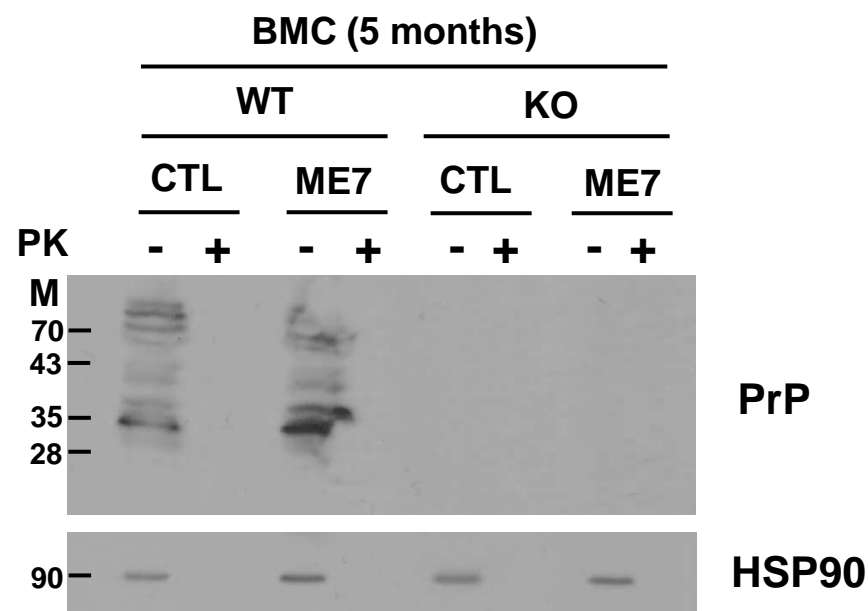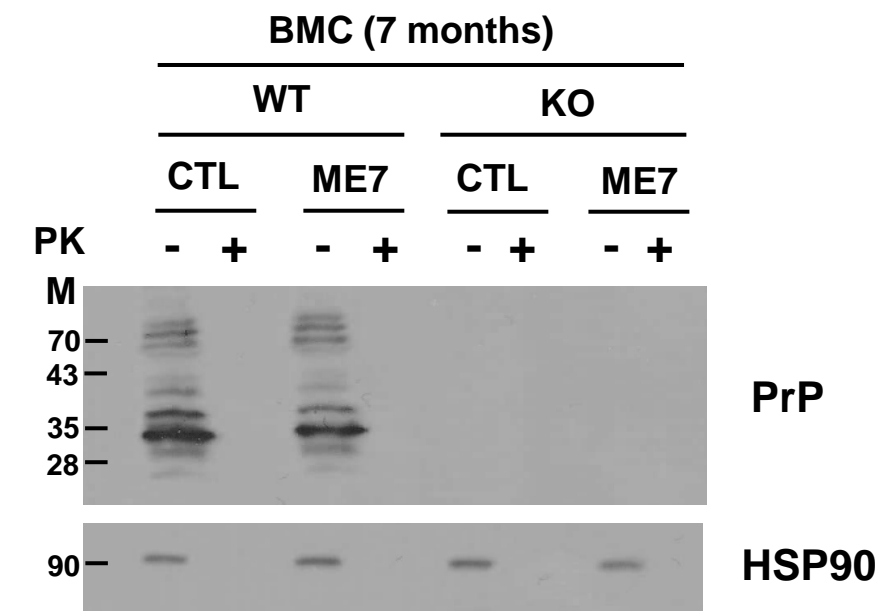

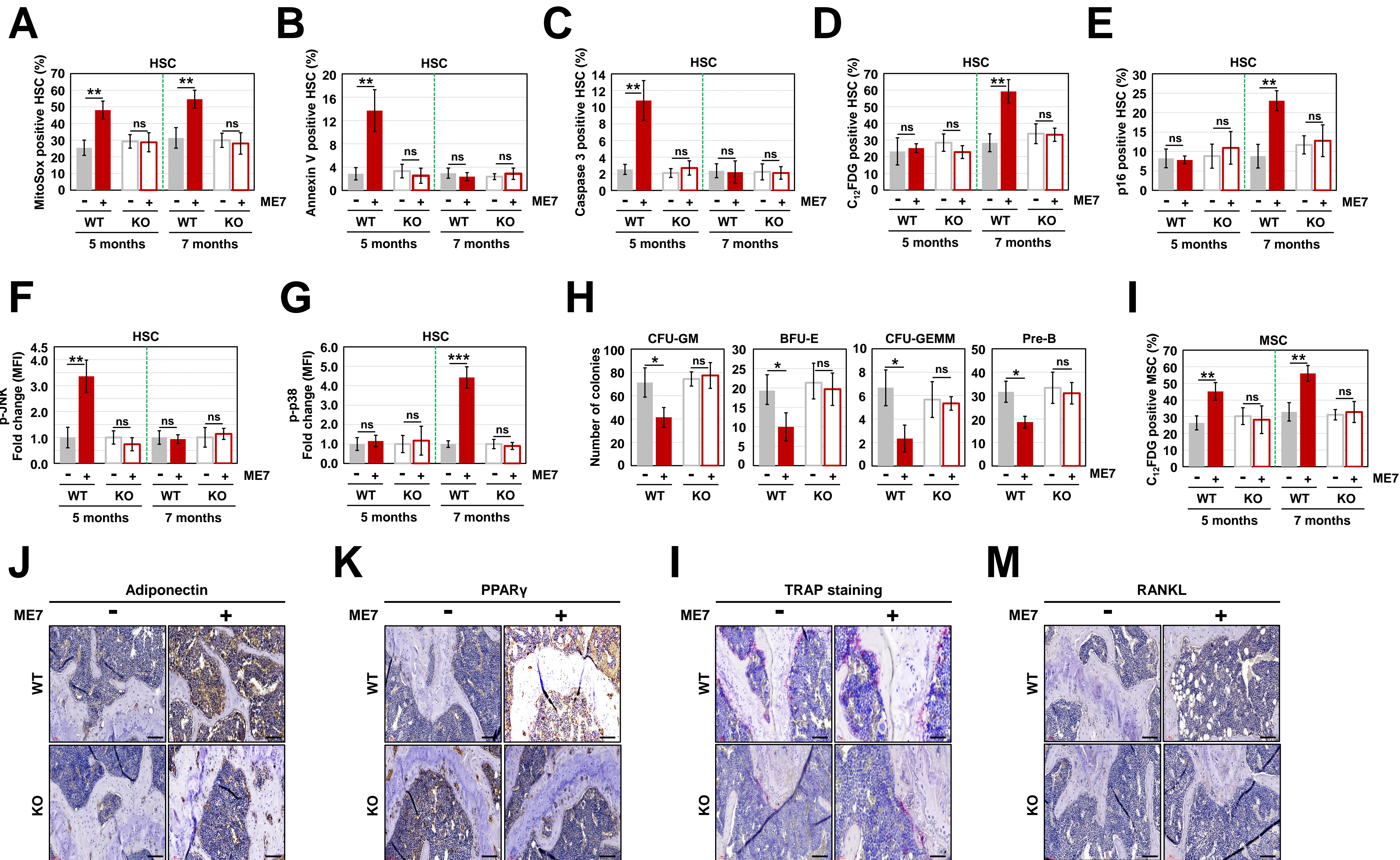

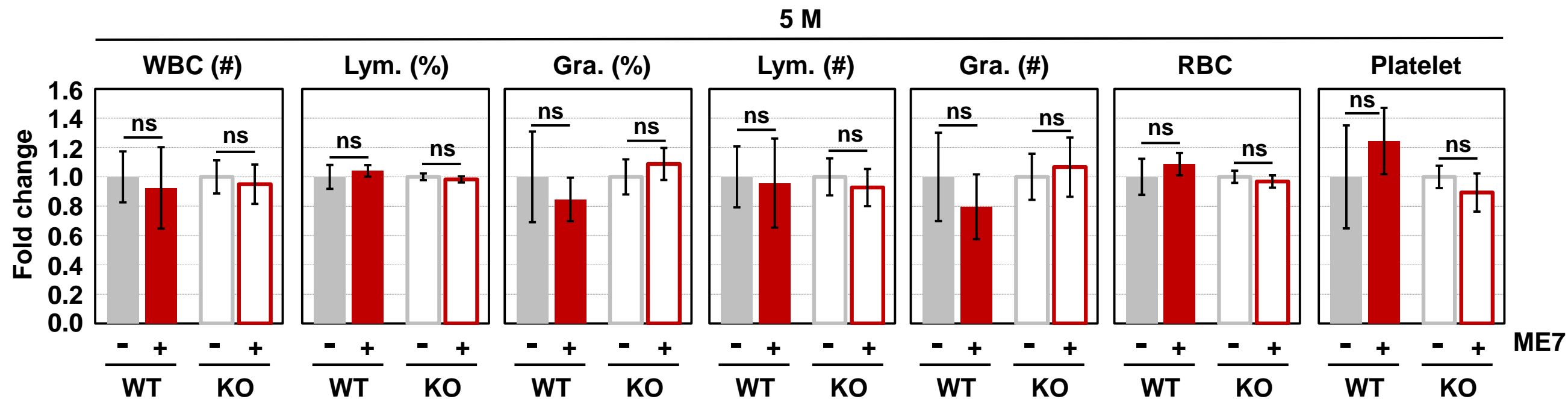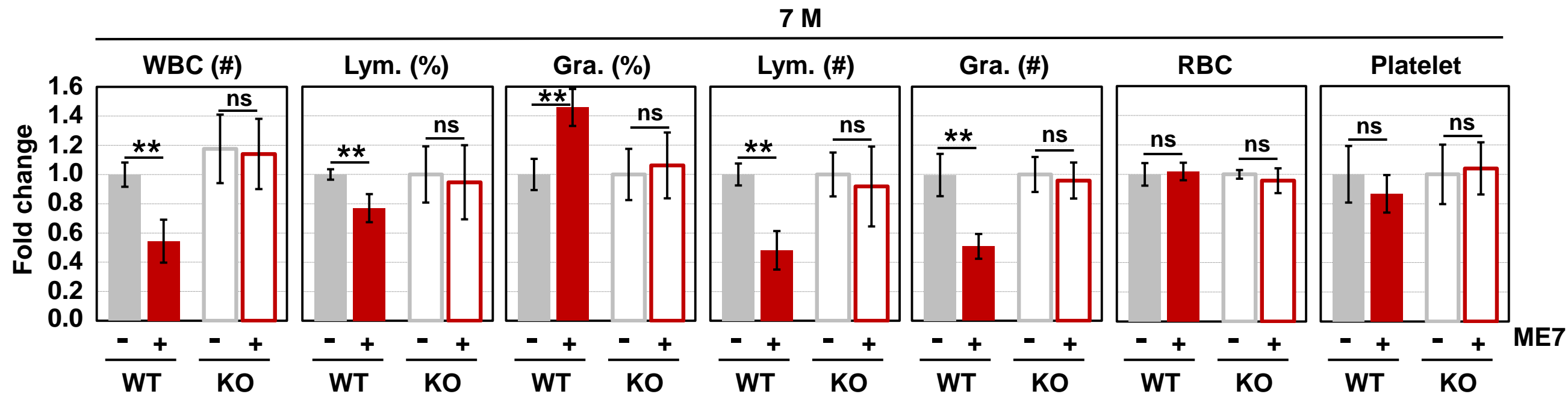

Supplement: Supplementary file 2 — Supplementary Figures [file 41375_2023_1828_MOESM2_ESM.pdf]
